# The Medical Library Association (MLA) voter: a survey of attitudes, perceptions, and voting practices in MLA national elections*

**DOI:** 10.5195/jmla.2020.480

**Published:** 2020-07-01

**Authors:** James Shedlock, Elizabeth Perkin McQuillen

**Affiliations:** 1 jshedlock@rcn.com, Retired MLA Member, Chicago, IL; 2 ebeth1800@gmail.com, Manager, Faculty Affairs, Support and Data, College of Nursing, Wayne State University, Detroit, MI

## Abstract

**Objective:**

Voting in professional associations is critical for selecting leaders who will implement a desirable vision for an association. Members of the Medical Library Association (MLA) were surveyed to assess their attitudes and perceptions of the voting process to elect the MLA national offices of president and members of the Board of Directors and Nominating Committee. Survey data were also used to test the hypothesis that committed MLA members are more likely to always vote.

**Methods:**

SurveyMonkey was used to deliver a 46-question survey to 2,671 email addresses of MLA members who were eligible to vote. Survey data were analyzed using quantitative and qualitative approaches.

**Results:**

A total of 676 responses were received, resulting in a 25% response rate. Respondents indicated that the most desired qualities in candidates included experience in professional positions, contributions to MLA, and a vision for the association, whereas candidates' personal characteristics were rarely considered. Respondents expressed doubts about the use of a single slate, had positive views of campaigning but were doubtful about its impact, and were generally accepting of the current voting process. Committed MLA members were significantly more likely to always vote in MLA national elections.

**Conclusions:**

The survey results provide insight into understanding the concerns and motivations of MLA voters and add to the limited literature on professional association voting.

**Figure:**
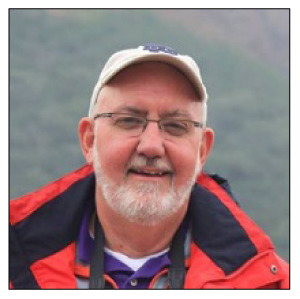
James Shedlock, AMLS, AHIP, FMLA

## INTRODUCTION

Voting, the formal expression of a choice between two or more issues or people [1], can take place in almost any group activity or social situation. By voting in civic elections, individuals exercise their democratic role to elect representative leaders who manage the affairs of the government. Similarly, members of professional associations vote to elect leaders who will carry out a vision for the association and, thereby, shape the future of their profession. As such, it is important to understand how the membership of professional associations views their associations' voting processes.

In the United States, civic voting participation tends to hover around 50% for presidential elections, which is lower than that in other nations [2]. Voting in professional associations also tends to be low, estimated at around 32% of membership rolls [3]. Medical Library Association (MLA) election return rates have ranged from 30%–40% of its membership over the last decade [4–8] (supplemental Appendix A).

A review of the literature revealed only two studies about professional association voting, both from the American Sociological Association (ASA), that could provide models for understanding low voting returns in other professional associations, such as MLA. In one ASA study, Ridgeway and Moore concluded that members who showed commitment to the organization were more likely to vote in association elections [9]. Later, D'Antonio and Tuch replicated Ridgeway and Moore's methods and conclusions using data from three presidential ASA elections and concluded that “members who participate in networks that link them, however directly or indirectly, to the larger Association are more inclined to vote than those not so linked” [10].

To assess MLA members' attitudes toward and perceptions of the voting process and to shed light on why some MLA members do not vote, voting-eligible members in the MLA were surveyed in 2017.

## METHODS

### Survey development and implementation

Based on his service on three MLA Nominating Committees (1991, 2000, and 2014), one author (Shedlock) created a survey using SurveyMonkey software consisting of 46 closed-ended and open-ended questions concerning respondents' demographics, frequency of voting in MLA national elections, attitudes toward and perceptions of the voting process, and perceived commitment or connection to MLA (supplemental Appendix B). The survey instrument was reviewed by a select group of colleagues who were highly experienced in the nominating and voting process to establish content validity. Cronbach's alpha for all scaled items was 0.72, indicative of acceptable internal validity. The survey was disseminated to all eligible MLA voters in January 2017 using 2,671 email addresses obtained from MLA headquarters staff. One follow-up email was sent to encourage more responses.

### Data analysis

SurveyMonkey generated a data file that scored item responses on a scale (1, “yes”; 2, “no”; 3, “not sure”; 0, blank) or other rating system, depending on the type of question; comments from open-ended questions were captured as text. When participants did not respond to an item, it was treated as missing data. In total, missing data accounted for less than 16% of all possible responses to survey items. Quantitative data analysis was performed using SPSS, version 2.5, and full quantitative survey results are provided in supplemental Appendix C. Comments to open-ended questions were analyzed by content analysis [11]. Coding was verified via two stages of analysis [12]: initial coding by the qualitative researcher using NVivo 11 Pro to create an initial coding structure, and independent coding by the author using a matrix technique [13]. Analysis of the open-ended comments was intended to further explain the quantitative survey results, with demonstrable evidence that the content analysis results were plausible, were cohesive, and corresponded with the close-ended survey responses [14, 15].

## RESULTS

When the survey closed in February 2017, 676 responses were received, resulting in a 25% response rate.

### Respondent demographics

Missing data, which did not appear to be systematic, were removed from analyses. Most respondents were women between 46–65 years of age (Table 1). Many had ≥26 years of experience in a library-related position, held a master's in library and information science degree (or variant) or were currently earning a masters' degree, and were employed full- or part-time in a library or similar type of information setting. Most held chapter or section membership. Most respondents were from the Midwest Chapter (19%), followed by the Mid-Atlantic Chapter (14%) and South Central Chapter (12%) (supplemental Appendix D). The lowest regional representation was from the Hawaii-Pacific Chapter (<1%). International members were from Canada (3%), St. Maarten (<1%), India (<1%), and China (<1%).

**Table 1:** Respondent demographics, employment, and membership

Demographic	n	%
Gender (n=676)		
Men	95	14%
Women	565	84%
No response	16	2%
Age range (n=661)		
≤25 years	2	0.3%
26–35 years	84	13%
36–45 years	122	18%
46–65 years	352	53%
≥66 years	101	15%
Years of experience in a library-related position (n=667)		
0–5 years	52	8%
6–10 years	94	14%
11–15 years	91	14%
16–20 years	76	11%
21–25 years	82	12%
≥26 years	272	41%
Degree (n=666)		
Master of library and information science degree (MLIS) or variant	571	86%
Doctorate (PhD) or doctor of education (EdD)	14	2%
Multiple graduate degrees	81	12%
Employment (n=660)		
Employed full- or part-time in a library or similar information setting	591	90%
Retired	60	9%
Other employment status	9	1%
Employment institution (n=608)		
Academia	364	60%
Hospital	165	27%
Other location	79	13%
MLA membership		
Chapter membership (n=6)	494	75%
Section membership (n=662)	500	76%

### Qualities and characteristics of candidates for MLA national offices

**Figure 1:**
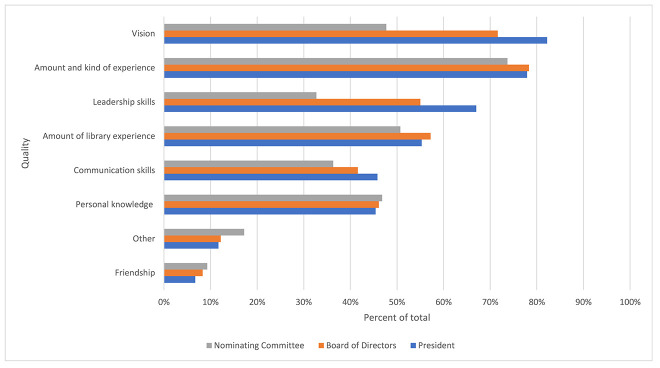
Desired qualities of candidates for Medical Library Association (MLA) national offices

Respondents were asked to indicate qualities that they look for in candidates for MLA national offices. Vision for MLA was the most important quality desired for presidential candidates (Figure 1). The amount and kind of MLA experience was the most desired quality for both Board of Directors and Nominating Committee candidates.

Respondents were then asked which other professional and personal characteristics they considered when deciding for whom to vote for an MLA national office. In terms of professional characteristics, a candidate's professional practice and work history was the most considered attribute, and their alma mater was the least considered (Table 2). Most personal characteristics (i.e., marital status, sexual orientation, race, gender) were rarely or never considered by voters. There was no significant association between the gender of the respondent and the frequency with which they considered the gender of candidates (χ^2^(5)=2.076, *p*=0.839).

**Table 2:** Frequency of consideration of candidates' professional and personal characteristics

	Always		Frequently		Sometimes		Rarely		Never		Total
	n	%	n	%	n	%	n	%	n	%	n
Professional practice and work history (predominant practice field; e.g., public services, technical services)	223	35%	225	35%	135	21%	33	5%	3	5%	645
Regional affiliation (i.e., geographic location where the candidate works or resides)	40	6%	118	18%	225	35%	94	15%	17	27%	640
Institutional affiliation (i.e., candidate's employer)	30	5%	104	16%	277	43%	101	16%	131	21%	638
Degree affiliation (i.e., candidate's alma mater)	9	1%	35	5%	96	15%	170	27%	335	53%	637
Gender	19	3%	49	8%	139	22%	131	20%	303	47%	640
Race	20	3%	50	8%	123	19%	79	12%	374	58%	642
Sexual orientation	15	2%	15	2%	40	6%	51	8%	514	81%	634
Marital status	0	-	1	0.1%	2	0.3%	29	5%	600	95%	631

When asked about the most important deciding factor when casting a vote for MLA president, respondents' open-ended responses suggested that a vision for MLA was the most deciding factor (129 out of 545 comments, 24%), followed by experience in MLA (95 comments, 17%), leadership and communication skills (57 comments, 10%), and personal knowledge of and/or some relationship with the candidate (34 comments, 6%). For Board of Directors candidates, experience in the profession or serving MLA was the most deciding factor, especially when that experience was gained in different library types or different geographic locations (95 out of 539 comments, 18%). Other responses highlighted vision for MLA (52 comments, 10%), personal knowledge of the candidate (36 comments, 7%), and leadership and communications skills (28 comments, 5%). For Nominating Committee candidates, MLA experience was the most deciding factor (81 out of 531 comments, 15%), followed by personal knowledge of the candidate (42 comments, 8%), diverse experience in library work and/or geographic locations (34 comments, 6%), vision and network connections (19 comments each, 4%), and communication skills (15 comments, 3%).

### Voting issues

#### Voting frequency.

When respondents were asked about the importance of voting every year for MLA national offices, approximately half (53%, n=331/626) said it was very important, 30% (n=186/626) said it was somewhat important, 12% (n=75/626) said it was slightly important, and 5% (n=34/626) said it was not at all important. When asked how frequently they vote in MLA national elections, slightly more than two-thirds of respondents said they always voted (68%, n=425/626), with fewer respondents saying they sometimes (20%, n=127/626), occasionally (9%, n=58/626), or never voted (3%, n=16/626). Some open-ended comments suggested why some members do not vote:

I haven't been a member long enough to establish a frequency. I will say it is difficult for new members when we don't know anyone. Sometimes the bios are not really specific to the position up for election.

Two factors: 1) I simply don't have time to keep abreast of what's happening at the national level…2) Until recently I worked at an institution that never budgeted travel money for me. I really couldn't participate in MLA in any meaningful way because I couldn't attend the national meetings. MLA became almost irrelevant to me for that reason.

#### Election of Nominating Committee.

MLA members elect a Nominating Committee to create a slate of candidates for national office, in contrast to other major library associations (e.g., American Library Association, Special Libraries Association, American Association of Law Libraries) that appoint nominating committees. When respondents were asked if they thought this MLA nomination process was useful, 53% (n=322/604) said that it is very useful, 43% (n=261/604) said it is moderately useful, and 3% (n=21/604) said it is not at all useful. One respondent commented, “Why do they have to be elected? I trust the Board to appoint an appropriate committee like they do all their other committees.” Other comments suggested that this process is confusing to many MLA members.

When asked if they had considered using “nomination by petition” to add additional candidates to the slate, 97% (n=595/615) of respondents said “no,” and 3% (n=20/615) said “yes.” A frequent comment for this question was, “I didn't know [this possibility] existed.” One respondent pointed out a problem with the petition process: “I usually only remember that we have elections when I get the ballot, and when I see the candidates, sometimes I wish I had another candidate to choose from. I never think ahead about candidates and wouldn't feel right petitioning.” When asked if they would consider using “nomination by petition” in the future, 58% said no (n=342/585), and 42% (n=243/585) said “yes.”

#### Single slate.

While the use of a single slate (i.e., a single candidate with no opposition) was last discussed at the 1999 MLA annual meeting [16], single slates have been used more frequently for section ballots. The survey assessed respondents' attitudes toward and perceptions of using a single slate, which would give the elected Nominating Committee responsibility for selecting MLA leadership. Most respondents (n=282/636, 44%) were not sure whether MLA should adopt a single slate for the president and Board of Directors, whereas 35% (n=226/636) were opposed to using a single slate, and 20% (n=128/636) were in favor. Respondents' comments reflected the dilemma of this issue:

I have served on the nominating committee and sometimes find it difficult to get good candidates, so it would be easier to only have to recruit one. I trust the nominating committee to make wise choices BUT I also think the membership should have choices[;] I am not sure I am ok either way.

I think it is something that should be investigated. We lose good talents because they run against someone, and then are not willing to run again.

Other respondents spoke for the value of a single slate:

In small organizations, contested slates are not necessary. This applies to MLA. Contested slates create hard feelings, dissent and disappointment and really must be detrimental to finding candidates at all to run for any office.

At the same time, many respondents were against the idea of using a single slate:

No, no, no, no, no! I don't even like it when my sections have a single slate!

It is important that MLA continue to allow members to have a role in the election process. I personally feel more connected and valued when I am actively engaged.

**Figure 2:**
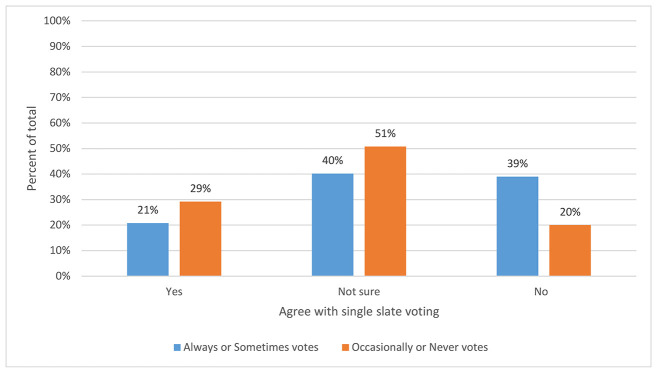
Association between voting frequency and opinion of a single slate*

In a closer analysis of responses concerning a single slate for MLA national elections, those who always or sometimes voted had significantly less favorable opinions of the use of a single slate than those who occasionally or never vote (*χ*^*2*^(2)=9.07, *p*=0.011; Figure 2).

• Single slate: Nominating Committee elects candidate.

While most respondents were not sure about using a single slate in MLA national elections, 68% (n=391/573) expressed no objection in open-ended responses to using single slates in sections or chapters. A strong minority of respondents (26%, n=148/573) were opposed to single slates in sections and chapters but accepted the practice. Only 34 of 573 respondents (6%) said they were opposed and did not vote in such elections.

#### Voting as a benefit versus responsibility.

**Figure 3:**
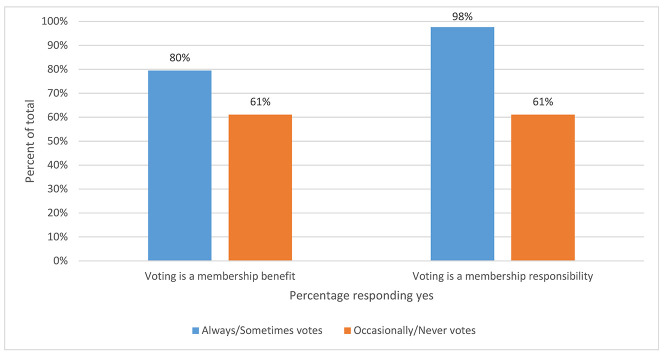
Association between voting frequency and view of voting as benefit or responsibility

Most respondents saw voting as both a membership benefit (n=482/622, 77%) and a membership responsibility (n=581/623, 93%). However, those who always or sometimes voted were significantly more likely to view voting as a benefit (χ^2^(1)=12.30, *p*<0.001) and a responsibility (χ^2^(1)=135.81, *p*<0.001; Figure 3).

#### Motivations for voting.

Respondents were asked what would encourage them to vote if they had not voted in most MLA elections. Most comments were about the need for more information about the candidates and their positions on challenges that MLA is facing (30 out of 129 comments, 23%). One respondent commented, “MLA offers CE via webinars. What about some sort of way to meet the candidates via a brief webinar with time for Q&A?” Others mentioned desiring more frequent reminders to vote (9 comments, 7%) and the need for and importance of attendance at the MLA annual meeting as a way to know the candidates (5 comments, 4%). One respondent mentioned the need to participate in MLA despite barriers that seem to stand in the way, such as not being selected to join a committee:

As a relatively new member,…I've been with MLA around 5–6 years…, I've never been able to participate in a committee or anything of the like despite applying every year…It feels difficult to vote for someone when you don't feel like they're concerned with or aware of your voice in the organization.

When asked whether they had ever nominated a person for MLA office, 80% (n=497/623) of respondents said “no,” and 20% (n=126/623) said “yes.” Of those who reported nominating a person for office, 72% (n=96/134) said their nominee was selected as a candidate. Most respondents (n=577/611, 94%) had not nominated themselves for MLA office, but of those who had (n=34/611, 6%), 37% (n=20/54) were selected as a candidate.

#### Candidate's statements.

Candidates for MLA office have primarily submitted statements in the *MLA News* (now, *MLAConnect*) following Nominating Committee guidelines (e.g., education credentials, professional experience, involvement in MLA, recent publications). Candidates respond to questions posed by the Nominating Committee as the chief means of stating their positions on a selected issue about the future of MLA, its direction, or a challenge deemed important by the committee. When asked if these candidate statements were useful in their decision-making process, 58% (n=359/616) of respondents said they were very useful, 34% (n=212/616) said they were somewhat useful, 6% (n=39/616) said they were rarely useful, and 1% (n=6) said they never read them.

#### Campaigning.

Traditionally, MLA has not used campaigning, in contrast to the common practice used by American Library Association presidential and treasurer candidates, who promote themselves and solicit votes through websites and emails. There has been no explicit statement by the MLA Board of Directors for or against this practice, and only recently (per the author's observation) has a candidate used social media for campaigning. Most (n=514/620, 83%) respondents said they had not engaged in campaigning on behalf of a candidate. Open-ended comments suggested that respondents did not engage in campaigning as an act of persuasion for or against a candidate but rather engaged with colleagues in conversation “about who might be the best choice.”

When asked whether MLA should consider using campaigning, most respondents said “yes” (n=329/622, 53%), but many (39%, n=245/622) expressed uncertainty about campaigning. When asked whether they thought campaigning would have a positive or negative impact on MLA elections, 55% (n=338/620) said they were not sure, 34% (n=213/620) said it would have a negative impact, and 11% (n=69/620) said it would have a positive impact. When asked if the MLA Board of Directors should define controls for campaigning, 48% (n=294/608) said “yes,” 42% (n=254/608) were not sure, and 10% (n=60/608) said “no.”

#### Candidates' advantages.

When asked what factors gave one candidate an advantage over another candidate in an MLA election, respondents said that experience in the profession and MLA was the best advantage (248 out of 482 comments, 51%). Other perceived advantages related to having name recognition (170 comments, 35%), a vision for MLA (59 comments, 12%), leadership skills (48 comments, 10%), a strong background mostly in an academic setting (33 comments, 7%), communication skills (14 comments, 3%), gender (10 comments, 2%), and diversity or race (7 comments, 1%). One respondent noted:

A past history of leadership and involvement in MLA is a big factor for me. I consider gender because the profession is predominantly made up of women and I want to be sure we don't have an abnormal proportion of men in MLA leadership roles. There are proportionally too many men in library leadership roles. Race: it's important to have more diversity in MLA leadership, which will also encourage more diversity in the membership.

### Relationship between involvement in MLA and voting

A series of survey questions around involvement in MLA were used to examine the hypothesis that active, committed members of MLA were more likely to vote in MLA national elections than members who were not as active. Commitment was defined as attendance at MLA annual and chapter meetings; service on a national committee, editorial board, ad hoc group, task force, jury, or other body; publishing in the *Journal of the Medical Library Association (JMLA)* or *MLAConnect*; or service to an MLA chapter or section (e.g., holding an elected office; service on or appointment to a committee, task force, group, or other body; publishing in a chapter or section publication).

Over half (n=341/617, 55%) of respondents reported commitments to MLA on a national level, and 35% (n=214/610) and 52% (n=317/611) reported commitments on a chapter or section level, respectively. Members who attended MLA annual meetings on a yearly basis (*χ*^*2*^(2)=16.20, *p*<0.001, n=524), attended MLA chapter meetings on a yearly basis (*χ*^*2*^(2)=20.30, *p*<0.001, n=524), and showed other forms of commitment to MLA on a national (*χ*^*2*^(1)=36.64, *p*<0.001, n=524), chapter (*χ*^*2*^(1)=49.93, *p*<0.001, n=524), or section (*χ*^*2*^(1)=36.80, *p*<0.001, n=524) level were significantly more likely to always or sometimes vote in MLA elections than to rarely or never vote.

In addition, logistic regression was performed to determine the degree to which attendance at MLA annual meetings or showing other forms of commitment at national, chapter, or section levels predicted whether respondents always voted. The model fit the data, as shown by a nonsignificant Hosmer and Lemeshow test (χ^2^(7)=5.13, *p*=0.644). The amount of variance explained by the model was 23% (Nagelkerke R^2^), and 72% of the cases were correctly classified. All commitment predictors, except for attending the MLA annual meeting every other year, were significantly associated with the likelihood of always voting (Table 3), indicating that they played meaningful roles in the frequency with which a member voted in MLA national elections. Per the odds ratio, individuals who attended the MLA annual meeting on a yearly basis were twice as likely to always vote than those who attended the annual meeting less frequently than every other year. Also, those who showed some form of commitment at national, chapter, or section levels were twice as likely to always vote than those who did not show this commitment.

**Table 3:** Results of logistic regression predicting the likelihood of always voting

Predictor	*B*	SE	Wald	*df*	*p*	Odds ratio	95% Confidence interval	
Attend MLA annual meeting yearly	0.76	0.23	10.52	1	0.001	2.14	1.35	3.38
Attend MLA annual meeting every other year	0.20	0.27	0.58	1	0.446	1.22	0.73	2.06
Current or past commitment on a national level	0.70	0.24	8.69	1	0.003	2.02	1.27	3.22
Current or past commitment on a chapter level	0.76	0.21	12.88	1	0.000	2.15	1.41	3.26
Current or past commitment on a section level	0.57	0.23	5.95	1	0.015	1.76	1.12	2.78

Note: Frequency of MLA annual meeting attendance compared with attending less than every other year and current or past service was compared with no current or past service. Chapter meeting attendance was highly correlated with annual meeting attendance and, therefore, was not included in the model.

#### Other connections to MLA.

In open-ended comments, respondents described feeling connected to MLA through other means than those mentioned in the survey. These means included the use of MLA resources (e.g., website, publications; 68 out of 199 comments, 34%); being active in MLA sections, special interest groups, and other units (38 comments, 19%); having personal friendships with colleagues and related networking opportunities (29 comments, 15%); and being active in chapters (19 comments, 10%). However, 10 respondents (5%) specifically mentioned their lack of connectedness to MLA.

#### Other issues.

The final survey question asked if there were other issues related to the MLA voting process that should be considered. Some respondents (10 out of 96 comments, 10%) mentioned the need to streamline the voting process. For example, “I feel that the whole Nominating Committee process was rather complicated,” “I think having chapters elect candidates to the Nominating Committee slate ensures election of a broad and representative national Nominating Committee that [is] able to identify capable and well qualified candidates,” and “[it would be nice to have] a FAQ link people can go to in order to familiarize themselves” about the current Nominating Committee process. Other respondents expressed the need to improve voter turnout (8 comments, 8%), the desire for a broader range of candidates (4 comments, 4%) or more diversity in candidate selection (3 comments, 3%), and the consideration of using video and not just text-based media in the voting process (3 comments, 3%).

## DISCUSSION

These survey results provided insight into understanding the concerns and motivations of MLA voters and added to the limited literature on professional association voting. These results provided a perspective on professional association voting from one corner of the library world: how North American medical librarians understood and perceived voting in MLA. Further research on how other national groups of health information professionals viewed voting would support a broader professional understanding of electing leadership positions.

This survey revealed the values that were important to MLA members when selecting their leaders: experience in professional work, contributions to MLA, and a vision for the association. While each Nominating Committee approaches their assignment of creating a national slate differently, experience suggests they approach the same question that MLA voters have utmost in their minds: what are the qualities of leadership that are wanted and needed as the association faces its future?

One outstanding theme was a desire for a more diverse leadership, which was not explicitly probed in the survey but was expressed by respondents in open-ended comments, indicating the importance of their concern. This diversity related to race and gender, different types of professional work or MLA contributions, and geographical location. As one respondent stated, “I think it's important to have a balance of genders, races, sexual orientations, and regional affiliations, especially for the Board of Directors and Nominating Committee.” Another stated, “voices from a range of perspectives [are] vital to the health of the organization.”

These survey results updated the MLA membership's view of an old voting issue—the use of a single slate for MLA national elections, which was last discussed in 1999—and suggested that MLA members are more doubtful about adopting this practice than in the past [17]. Such doubts about adopting a single slate could be due to respondents' experience with using a single slate for some sections and chapters in addition to a realization that MLA was getting smaller in size, making the use of a single slate an option to consider.

The survey was also used to explore a new voting topic: campaigning for office via social media. The survey showed some interest in its use as well as some ambivalence. Respondents' comments about campaigning emphasized the value of having more information about candidates available, and the use of social media or similar technology could meet this need. Ideally, the controlled use of social media could enlighten the membership about candidates' experiences and vision for MLA and personalize candidates so voters could know them “up close and personal” before they cast their votes. In other words, campaigning could be a moot issue if there were better means of informing the membership about candidates for national office.

The survey suggested that the MLA membership viewed the current MLA national voting system positively, namely the election of a Nominating Committee and reliance on candidates' statements published in *MLAConnect* prior to the election. This acceptance of the present MLA voting process also confirmed a conclusion drawn by D'Antonio and Tuch [10]: that a passive acceptance of the status quo might explain why members chose not to vote.

This survey had some limitations. Topics, such as a single slate, might have needed to be better defined for members who might not be familiar with the practice. Also, surveys such as this one might not do well in understanding the views of nonvoters, particularly for questions such as whether voting was considered a benefit of membership or a responsibility. Thus, developing a survey that specifically targeted nonvoters could allow more concrete conclusions about how best to increase MLA election return rates. Another improvement would be to pretest the survey with a larger, more diverse population.

Using previous studies from the ASA voting as models, one of the goals of the survey was to investigate why MLA members did not vote. The hypothesis, which was confirmed by this analysis, was that MLA members voted due to their connectedness to or participation in the MLA organization. Thus, members who volunteered and participated in the activities of the association sought to fulfill their responsibility as a full member of the association: I join, I participate, therefore I vote.

## SUPPLEMENTAL FILES

**Appendix A:** Medical Library Association voting return rate

**Appendix B:** Voter survey instrument

**Appendix C:** Quantitative survey results

**Appendix D:** Respondents' geographic location by Medical Library Association chapter
