# Supplementary material for: The Medical Library Association (MLA) voter: a survey of attitudes, perceptions, and voting practices in MLA national elections
Source: J Med Libr Assoc. 2020 Jul 1;108(3):452–62. doi: 10.5195/jmla.2020.480 (PMC7441894; doi:10.5195/jmla.2020.480)
Supplement: Supplementary file 1 — Appendix A: Medical Library Association voting return rate [file jmla-108-3-452-s01.pdf]

JMLA Journal of the Medical Library Association

Supplemental content to
J Med Libr Assoc. Jul;108(3):dx.doi.org/10.5195/jmla.2020.480
www.jmla.mlanet.org
© Shedlock, McQuillen 2020

The Medical Library Association (MLA) voter: a survey of attitudes, perceptions, and voting practices in MLA national elections

James Shedlock, AMLS, AHIP, FMLA; Elizabeth Perkin McQuillen, PhD

APPENDIX A
Medical Library Association voting return rate

Year	Medical Library Association voting return rate
2016–2017	31.58%
2015–2016	43.92%
2013–2014	40.26%
2011–2012	37.65%
2010–2011	37.52%
2009–2010	39.75%
