# Supplementary material for: The Medical Library Association (MLA) voter: a survey of attitudes, perceptions, and voting practices in MLA national elections
Source: J Med Libr Assoc. 2020 Jul 1;108(3):452–62. doi: 10.5195/jmla.2020.480 (PMC7441894; doi:10.5195/jmla.2020.480)
Supplement: Supplementary file 2 — Appendix B: Voter survey instrument [file jmla-108-3-452-s02.pdf]

JMLA Journal of the Medical Library Association

Supplemental content to
J Med Libr Assoc. Jul;108(3):dx.doi.org/10.5195/jmla.2020.480
www.jmla.mlanet.org
© Shedlock, McQuillen 2020

The Medical Library Association (MLA) voter: a survey of attitudes, perceptions, and voting practices in MLA national elections

James Shedlock, AMLS, AHIP, FMLA; Elizabeth Perkin McQuillen, PhD

APPENDIX B
Voter survey instrument

Voting in civic elections is considered the highest responsibility of citizenship. In professional associations, voting is also the highest responsibility of membership. Professional association voting gives members a voice in selecting the association's leadership and shaping the direction of the profession. Through voting, individuals exercise and fulfill their responsibility to the association and the profession.

The purpose of this research project is to study the MLA membership's perception of the voting process for MLA national office (president, Board of Directors candidates, Nominating Committee candidates), to study the preferred qualities of the candidates running for office and reasons for not voting, and to understand related voting attitudes and practices in MLA's national elections.

This project's motivation comes from the observation that, generally, only a third of the MLA membership votes in MLA's national elections for president, Board of Directors candidates, and Nominating Committee candidates. This survey explores why this is so by testing the hypothesis that members who are more involved in the association are the ones most likely to vote and members who are not involved tend to vote infrequently or not at all. Involvement includes such factors as currently or previously holding elected office at the national, section, or chapter level; currently or previously holding a task force or committee assignment; having one or more section memberships; having a chapter membership; frequently attending national or chapter meetings; writing for association publications and/or presenting at profession-related conferences and meetings; and so on.

Outcomes of this project will inform the membership and provide data for future MLA candidates and Nominating Committees.

1. Indicate the MLA region where you work and/or reside:

O Hawaii-Pacific Chapter
O Medical Library Group of Southern California and Arizona (MLGSCA)
O Mid-Atlantic Chapter (MAC)
O Midcontinental Chapter (MCMLA)
O Midwest Chapter
O New York-New Jersey Chapter (NY-NJ)
O North Atlantic Health Sciences Libraries (NAHSL)
O Northern California and Nevada Medical Library Group (NCNMLG)
O Pacific Northwest Chapter of MLA (PNC)
O Philadelphia Regional Chapter
O South Central Chapter (SCC)
O Southern Chapter
O Upstate New York and Ontario Chapter (UNYOC)
O Canada

Other country (please specify)

Supplemental content to
J Med Libr Assoc. Jul;108(3):dx.doi.org/10.5195/jmla.2020.480
www.jmla.mlanet.org
© Shedlock, McQuillen 2020

2. MLA membership: Mark one membership category.
- O Individual/personal (includes Regular, Life, Fellow, Emeritus, Student)
- O Institutional
- O Both personal and institutional
- O Other: Affiliate, International, Honorary

3. MLA membership: Do you also hold an MLA chapter membership?
- O Yes
- O No

4. MLA membership: Do you also hold an MLA section membership?
- O Yes
- O No

5. MLA membership: If you hold an MLA section membership, how many sections do you belong?
- O One
- O Two
- O More than two

6. What is your gender?
- O Female
- O Male

7. What is your age?
- O under 25 years
- O 26–35 years
- O 36–45 years
- O 46–65 years
- O 66–more years

8. Years of experience: Indicate your number of years working (or have worked in) library-related position(s).
- O 0–5 years
- O 6–10 years
- O 11–15 years
- O 16–20 years
- O 21–25 years
- O 26–30+ years

9. What professional degree(s) do you have?
- O Master of library/information science (MLIS or variant) (or currently earning a masters' degree)
- O Other master's degree
- O Doctorate (PhD)
- O Other degree(s) or professional certificates (please specify). Use this block to indicate your field of study if you possess a PhD. List other master's degrees beside or in place of the MLIS.

Supplemental content to
J Med Libr Assoc. Jul;108(3):dx.doi.org/10.5195/jmla.2020.480
www.jmla.mlanet.org
© Shedlock, McQuillen 2020

10. Your current work status:
- o Student (currently working full-time toward a MLIS degree or its variant) (Mark here if you are working in a paid or credit internship related to your MLIS course work).
- o Student-employee (mark here if you are working toward a MLIS degree and working in a paid, full- or part-time position)
- o Employed, full or part-time in a library/information setting or environment
- o Unemployed (seeking work)
- o Retired from library work (no longer working or working as an unpaid volunteer or working in another field)

Other (please specify)

---

11. Institution: If currently employed (including as a student intern or student employee [see above question], in what type of health sciences librarian are you? (Skip if not currently employed.)
- o Hospital librarian
- o Academic librarian
- o Special health librarian (working in a corporate environment, a not-for-profit organization, health association, etc.)

Other (please specify)

---

12. What qualities do you look for when voting for MLA president? Check all that apply.
- o Amount and kind of MLA experience (i.e., service on national committees, task forces, etc.; service to MLA sections and chapters; etc.)
- o Amount of library experience
- o Leadership skills
- o Vision for MLA and the profession expressed through candidate's statement, publications, presentations, etc.
- o Communication skills
- o Personal knowledge of the candidate
- o Friendship with the candidate
- o Other (please specify) ______________________________

13. What qualities do you look for when voting for MLA Board of Directors? Check all that apply.
- o Amount and kind of MLA experience (i.e., service on national committees, task forces, etc.; service to MLA sections and chapters; etc.)
- o Amount of library experience
- o Leadership skills
- o Vision for MLA and the profession expressed through candidate's statement, publications, presentations, etc.
- o Communication skills
- o Personal knowledge of the candidate
- o Friendship with the candidate
- o Other (please specify) ______________________________

JMLA Journal of the Medical Library Association

SC
Supplemental content to
J Med Libr Assoc. Jul;108(3):dx.doi.org/10.5195/jmla.2020.480
www.jmla.mlanet.org
© Shedlock, McQuillen 2020

14. What qualities do you look for when voting for MLA Nominating Committee? Check all that apply.
- O Amount and kind of MLA experience (i.e., service on national committees, task forces, etc.; service to MLA sections and chapters; etc.)
- O Amount of library experience
- O Leadership skills
- O Vision for MLA and the profession expressed through candidate's statement, publications, presentations, etc.
- O Communication skills
- O Personal knowledge of the candidate
- O Friendship with the candidate
- O Other (please specify) ______________________________

15. Given all the factors above that are important to you when voting for MLA national office (president, Board of Directors, Nominating Committee) and given that these factors are relatively equal among the candidates, do you consider any of the following characteristics when making a decision for whom to vote?

	**Always**	**Frequently**	**Sometimes**	**Rarely**	**Never**
Gender	O	O	O	O	O
Race	O	O	O	O	O
Sexual orientation	O	O	O	O	O
Marital status	O	O	O	O	O
Institutional affiliation (the candidate's employer)	O	O	O	O	O
Regional affiliation (the geographic area where the candidate works or is from)	O	O	O	O	O
Degree affiliation (the candidate's alma mater)	O	O	O	O	O
Professional practice and work history (the candidate's predominant practice field; i.e., public services, technical services, media services, technology services, administrative services, etc.)	O	O	O	O	O

Other factors or characteristics (please specify)

____

16. Given all the factors above – professional and personal – what is the *one or most important deciding factor* when casting your vote for MLA president?

____

17. Given all the factors above – professional and personal – what is the *one or most important deciding factor* when casting your vote for MLA Board of Directors?

____

Supplemental content to
J Med Libr Assoc. Jul;108(3):dx.doi.org/10.5195/jmla.2020.480
www.jmla.mlanet.org
© Shedlock, McQuillen 2020

18. Given all the factors above—professional and personal—what is the *one or most important deciding factor* when casting your vote for a candidate for Nominating Committee?

---

19. Do you believe MLA should use a single slate for president and Board of Directors (i.e., rely on the elected Nominating Committee to find the right leadership for MLA)?
O Yes
O No
O Not sure
Comment

---

20. What is your view of single slates for sections and/or chapters?
O Opposed and do not vote
O Opposed but accept the practice
O Find no objection
Comment

---

21. How important is it to vote every year for MLA leadership positions (president, Board of Directors, Nominating Committee)?
O Not at all important
O Slightly important
O Somewhat important
O Very important
Comment

---

22. Mark the statement most true to your experience:
O I always vote in MLA's national election.
O I sometimes vote in MLA elections (3 or more times out of every 5 elections).
O I occasionally vote in MLA elections (2 or fewer times out of 5 elections).
O I never vote in MLA elections.
If you never vote in MLA national elections, please explain why.

---

23. If you haven't voted in most MLA national elections, what would encourage you to vote in an MLA national election?

---

24. Do you consider voting in MLA elections a membership benefit?
O Yes
O No
If No, what benefit is more important?

Supplemental content to
J Med Libr Assoc. Jul;108(3):dx.doi.org/10.5195/jmla.2020.480
www.jmla.mlanet.org
© Shedlock, McQuillen 2020

25. Do you consider voting in MLA elections a membership responsibility?
- o Yes
- o No

Comment

---

26. If you have both individual/personal and institutional memberships, do you ever split your vote among candidates (i.e., use personal membership to vote for one candidate and institutional membership to vote for another candidate)?
- o Routinely
- o Sometimes
- o Rarely
- o Never
- o Not applicable

Comment

---

27. Have you ever nominated a person for MLA office?
- o Yes
- o No

28. If Yes was marked in Q 27, was the person selected as a candidate?
- o Yes
- o No

29. Have you ever nominated yourself for MLA office?
- o Yes
- o No

30. If Yes was selected in Q 29, were you selected as a candidate?
- o Yes
- o No

31. Have you ever "campaigned" on behalf of an MLA nominee? Campaigning is defined here as conversing with MLA members inside your library, in your geographic region or chapter, in a section, among friends in MLA, etc., for the purpose of persuading a vote on behalf of or against an MLA nominee. Conversing may be in person, by phone, via email, or via other social media.
- o Yes
- o No

Comment

---

32. Traditionally, MLA has not engaged in campaign activities for national office. Do you agree with this tradition?
- o Yes
- o No
- o Not sure

Please explain. If yes, why? If no, why?

JMLA Journal of the Medical Library Association

SC
Supplemental content to
J Med Libr Assoc. Jul;108(3):dx.doi.org/10.5195/jmla.2020.480
www.jmla.mlanet.org
© Shedlock, McQuillen 2020

33. Do you think campaigning would have a positive or negative impact on MLA elections?
O Positive
O Negative
O Not sure
Please share any thoughts and opinions you have about campaigning for MLA elections.

---

34. Do you think the MLA Board should define controls for campaigning in MLA national elections?
O Yes
O No
O Not sure
If yes, what criteria would you recommend for MLA campaigning?

---

35. How useful are candidate's statements in your decision-making process?
O Very useful
O Somewhat useful
O Rarely useful
O I never read them
Comment

---

36. How useful is MLA's Nominating Committee process (i.e., electing a Nominating Committee to create a slate of candidates for president and Board of Directors and asking the candidates questions about their views for the office they agree to seek)?
O Very useful
O Moderately useful
O Not at all useful
If the Nominating Committee process is not useful, can you suggest an alternative?

---

37. Have you considered nominating an additional candidate to the Nominating Committee's slate (i.e., using the nomination by petition process)?
O Yes
O No
Any comments about the nominating petition process?

---

38. Would you consider using the nomination by petition process in the future?
O Yes
O No
Comment

---

39. In your opinion, what factor(s) gives an MLA candidate an advantage over another candidate?

JMLA Journal of the Medical Library Association

SC
Supplemental content to
J Med Libr Assoc. Jul;108(3):dx.doi.org/10.5195/jmla.2020.480
www.jmla.mlanet.org
© Shedlock, McQuillen 2020

40. How often do you attend MLA annual meetings?
- Every year
- Every other year
- Fewer than every other year

41. How often do you attend chapter meetings?
- Every year
- Every other year
- Fewer than every other year

42. Are you currently on or have you ever served on a *national* committee, editorial board, ad hoc group, task force, jury, or other national body, or written for a national publication (*JMLA/BMLA, MLA News*)?
- Yes
- No

43. Are you currently on or have you ever served your MLA *chapter* via holding an elected office; serving on a chapter committee; having been appointed to a committee, task force, group, or other body; or having written for a chapter publication; etc.?
- Yes
- No

44. Are you currently on or have you ever served an MLA *section* via holding an elected office; serving on a section committee; having been appointed to a committee, task force, group, or other body; or having written for a section publication; etc.?
- Yes
- No

45. Please offer any other ways you feel connected to MLA, its chapters, and its sections.

---

46. Are there other issues related to the MLA voting process that should be considered?
