# Supplementary material for: The Medical Library Association (MLA) voter: a survey of attitudes, perceptions, and voting practices in MLA national elections
Source: J Med Libr Assoc. 2020 Jul 1;108(3):452–62. doi: 10.5195/jmla.2020.480 (PMC7441894; doi:10.5195/jmla.2020.480)
Supplement: Supplementary file 3 — Appendix C: Quantitative survey results [file jmla-108-3-452-s03.pdf]

JMLA Journal of the Medical Library Association

Supplemental content to
J Med Libr Assoc. Jul;108(3):dx.doi.org/10.5195/jmla.2020.480
www.jmla.mlanet.org
© Shedlock, McQuillen 2020

The Medical Library Association (MLA) voter: a survey of attitudes, perceptions, and voting practices in MLA national elections

James Shedlock, AMLS, AHIP, FMLA; Elizabeth Perkin McQuillen, PhD

APPENDIX C
Quantitative survey results

Q1 Indicate the MLA region where you work and/or reside:

Answered: 667 Skipped: 9

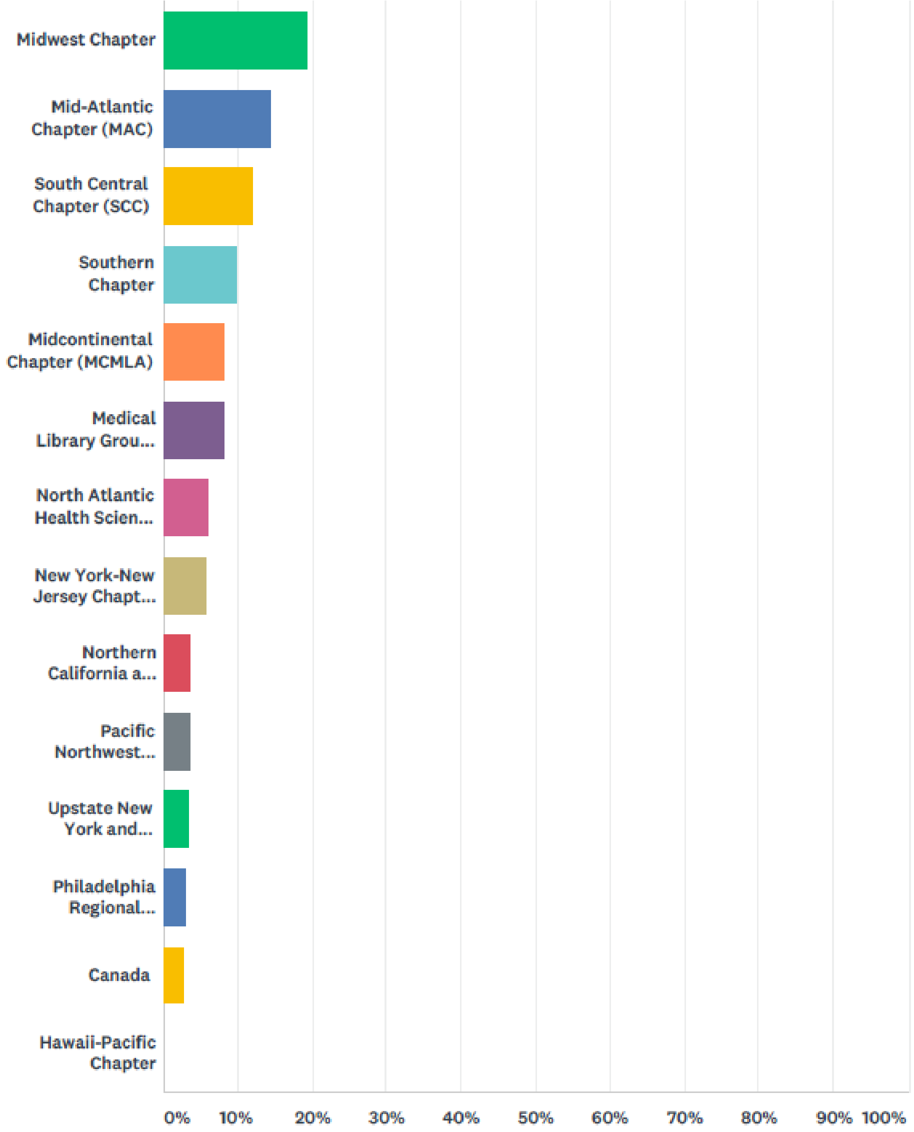

Supplemental content to
J Med Libr Assoc. Jul;108(3):dx.doi.org/10.5195/jmla.2020.480
www.jmla.mlanet.org
© Shedlock, McQuillen 2020

Answer choices	Responses	
Midwest Chapter	19.19%	128
Mid-Atlantic Chapter (MAC)	14.39%	96
South Central Chapter (SCC)	11.99%	80
Southern Chapter	9.90%	66
Midcontinental Chapter (MCMLA)	8.25%	55
Medical Library Group of Southern California and Arizona (MLGSCA)	8.10%	54
North Atlantic Health Sciences Libraries (NAHSL)	6.00%	40
New York-New Jersey Chapter (NY-NJ)	5.85%	39
Northern California and Nevada Medical Library Group (NCNMLG)	3.60%	24
Pacific Northwest Chapter of MLA (PNC)	3.60%	24
Upstate New York and Ontario Chapter (UNYOC)	3.30%	22
Philadelphia Regional Chapter	3.00%	20
Canada	2.70%	18
Hawaii-Pacific Chapter	0.15%	1
Total		667

Q2 MLA membership: Mark one membership category.
Answered: 667 Skipped: 9

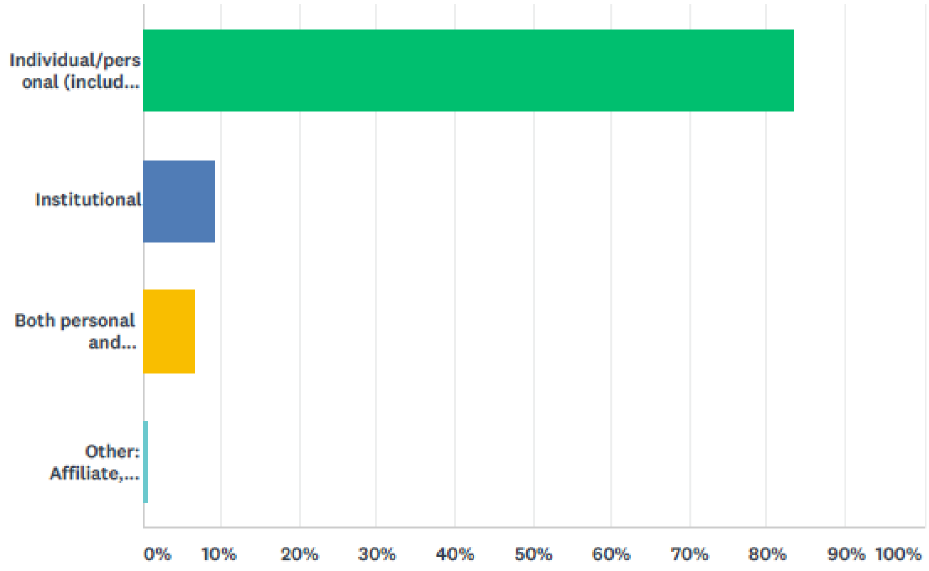

JMLA Journal of the Medical Library Association

SC
Supplemental content to
J Med Libr Assoc. Jul;108(3):dx.doi.org/10.5195/jmla.2020.480
www.jmla.mlanet.org
© Shedlock, McQuillen 2020

Answer choices	Responses	
Individual/personal (includes regular, life, fellow, emeritus, student)	83.36%	556
Institutional	9.30%	62
Both personal and institutional	6.75%	45
Other: Affiliate, International, Honorary	0.60%	4
Total		667

Q3 MLA membership: Do you also hold an MLA chapter membership?
Answered: 658 Skipped: 18

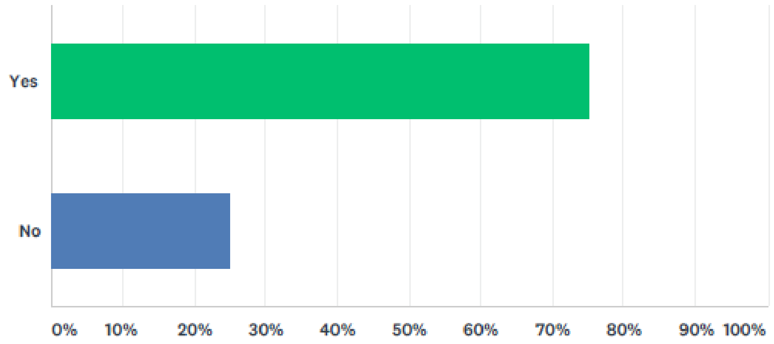

Answer choices	Responses	
Yes	75.08%	494
No	24.92%	164
Total		658

Q4 MLA membership: Do you also hold an MLA section membership?
Answered: 662 Skipped: 14

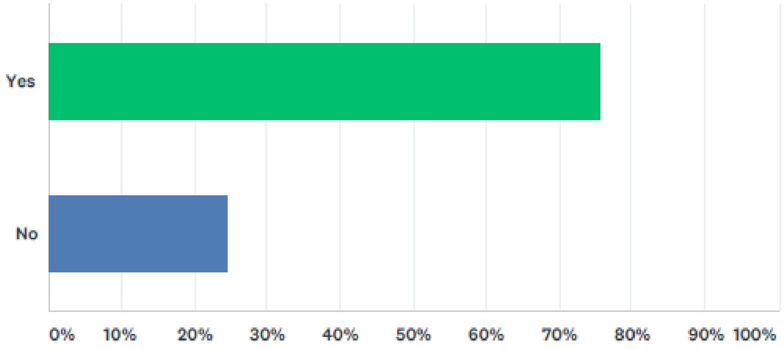

JMLA Journal of the Medical Library Association

SC
Supplemental content to
J Med Libr Assoc. Jul;108(3):dx.doi.org/10.5195/jmla.2020.480
www.jmla.mlanet.org
© Shedlock, McQuillen 2020

Answer choices	Responses	
Yes	75.53%	500
No	24.47%	162
Total		662

Q5 MLA membership: If you hold an MLA section membership, how many sections do you belong?
Answered: 505 Skipped: 171

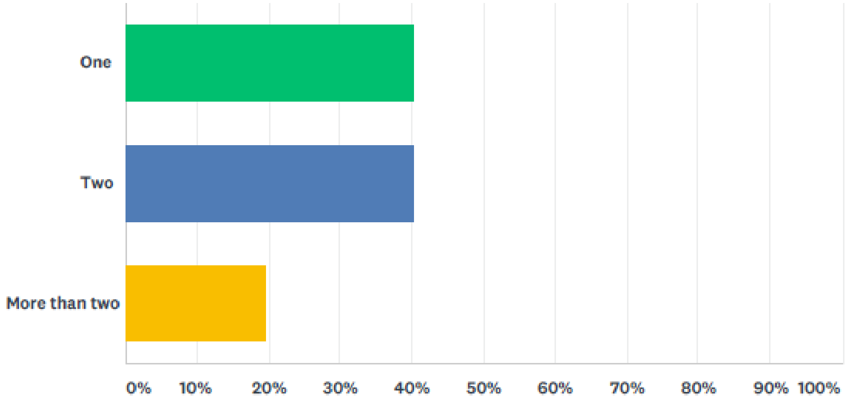

Answer choices	Responses	
One	40.20%	203
Two	40.20%	203
More than two	19.60%	99
Total		505

Q6 What is your gender?
Answered: 660 Skipped: 16

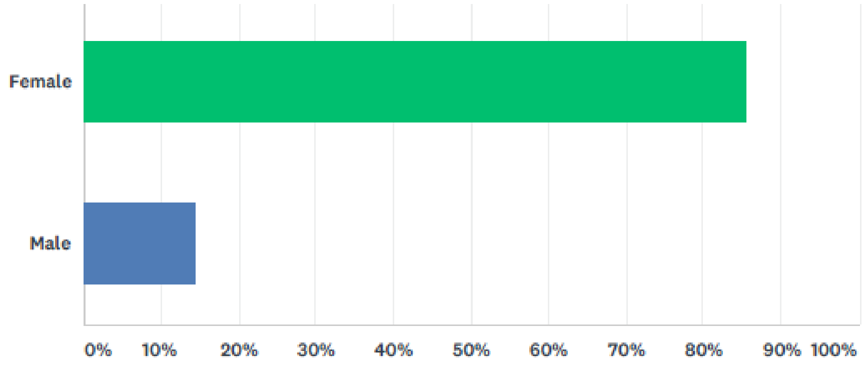

JMLA Journal of the Medical Library Association

SC
Supplemental content to
J Med Libr Assoc. Jul;108(3):dx.doi.org/10.5195/jmla.2020.480
www.jmla.mlanet.org
© Shedlock, McQuillen 2020

Answer choices	Responses	
Female	85.61%	565
Male	14.39%	95
Total		660

Q7 What is your age?
Answered: 661 Skipped: 15

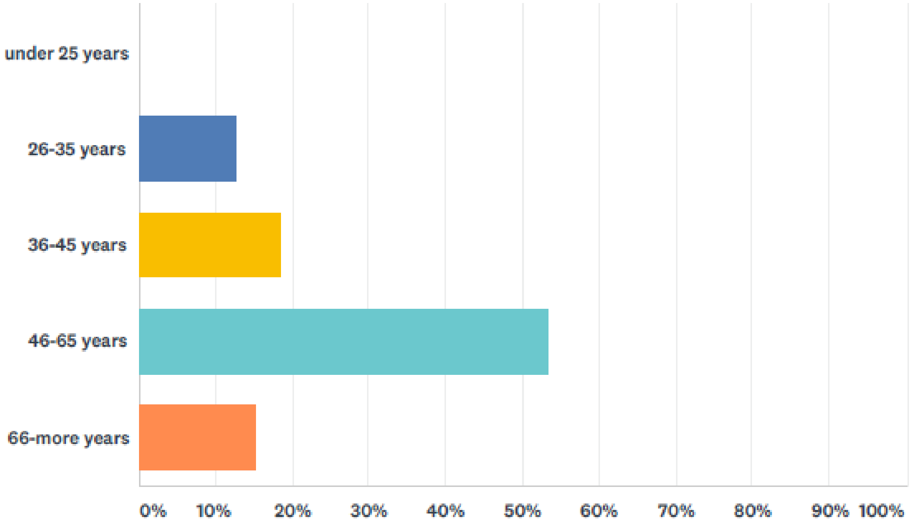

Answer choices	Responses	
under 25 years	0.30%	2
26–35 years	12.71%	84
36–45 years	18.46%	122
46–65 years	53.25%	352
66–more years	15.28%	101
Total		661

Supplemental content to
J Med Libr Assoc. Jul;108(3):dx.doi.org/10.5195/jmla.2020.480
www.jmla.mlanet.org
© Shedlock, McQuillen 2020

Q8 Years of experience: Indicate your number of years working (or have worked in) library-related position(s).

Answered: 667 Skipped: 9

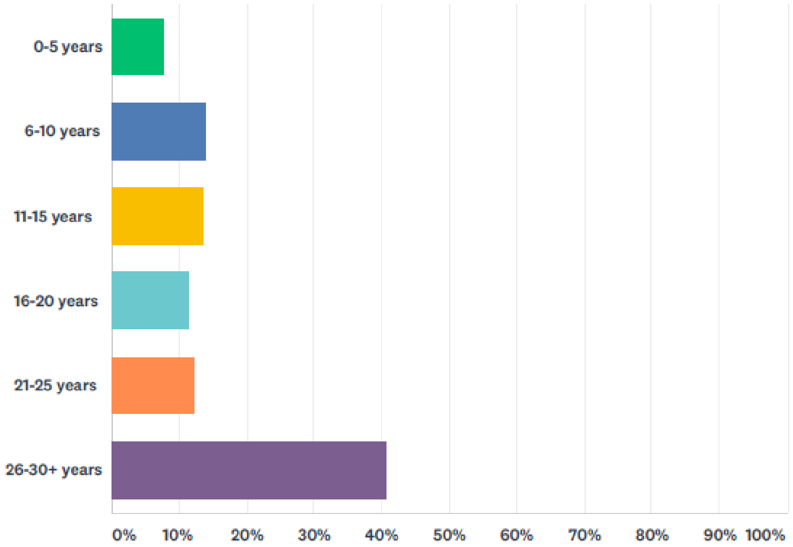

Answer choices	Responses	
0–5 years	7.80%	52
6–10 years	14.09%	94
11–15 years	13.64%	91
16–20 years	11.39%	76
21–25 years	12.29%	82
26-30+ years	40.78%	272
Total		667

Q9 What professional degree(s) do you have?
Answered: 666 Skipped: 10

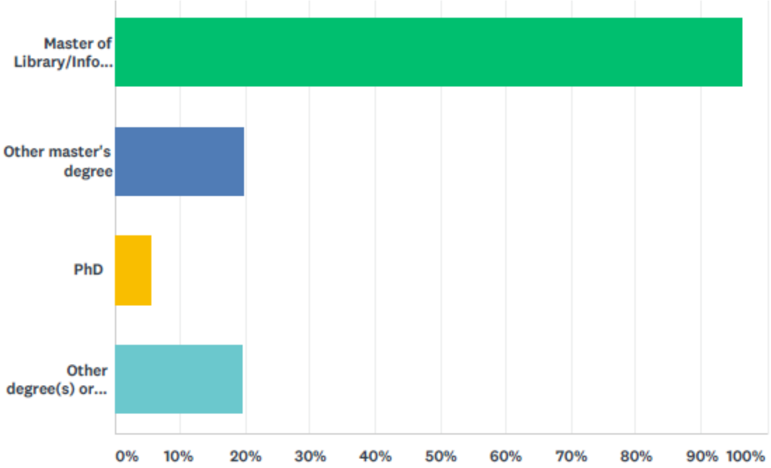

Answer choices	Responses	
Master of library/information science (MLIS or variant) (or currently earning a masters' degree)	96.25%	641
Other master's degree	19.97%	133
Doctorate (PhD)	5.71%	38
Other degree(s) or professional certificates (please specify). Use this block to indicate your field of study if you possess a PhD. List other master's degrees beside or in place of the MLIS.	19.67%	131
Total		666

Q10 Your current work status:
Answered: 660 Skipped: 16

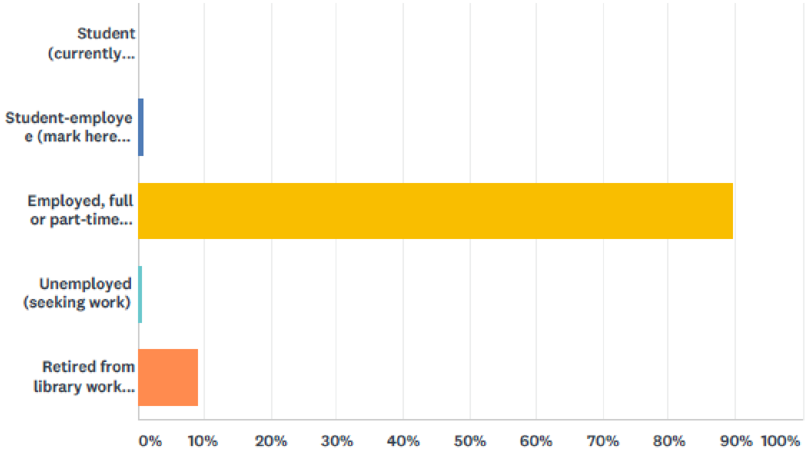

JMLA Journal of the Medical Library Association

Supplemental content to
J Med Libr Assoc. Jul;108(3):dx.doi.org/10.5195/jmla.2020.480
www.jmla.mlanet.org
© Shedlock, McQuillen 2020

Answer choices	Responses	
Student (currently working full-time toward a MLIS degree or its variant) (Mark here if you are working in a paid or credit internship related to your MLIS course work).	–	0
Student-employee (mark here if you are working toward a MLIS degree and working in a paid, full- or part-time position)	0.76%	5
Employed, full or part-time in a library/information setting or environment	89.55%	591
Unemployed (seeking work)	0.61%	4
Retired from library work (no longer working or working as an unpaid volunteer or working in another field)	9.09%	60
Total		660

Q11 Institution: If currently employed (including as a student intern or student employee [see above question], what type of health sciences librarian are you? (Skip if not currently employed.)

Answered: 608 Skipped: 68

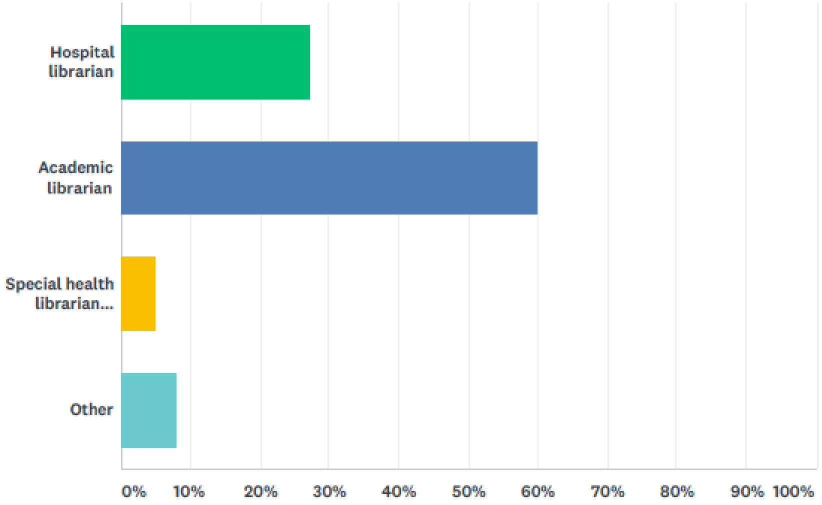

Answer choices	Responses	
Hospital librarian	27.14%	165
Academic librarian	59.87%	364
Special health librarian (working in a corporate environment, a not-for-profit organization, health association; etc.)	5.10%	31
Other (please specify)	7.89%	48
Total		608

JMLA Journal of the Medical Library Association

SC
Supplemental content to
J Med Libr Assoc. Jul;108(3):dx.doi.org/10.5195/jmla.2020.480
www.jmla.mlanet.org
© Shedlock, McQuillen 2020

Q12 What qualities do you look for when voting for MLA president? Check all that apply.
Answered: 666 Skipped: 10

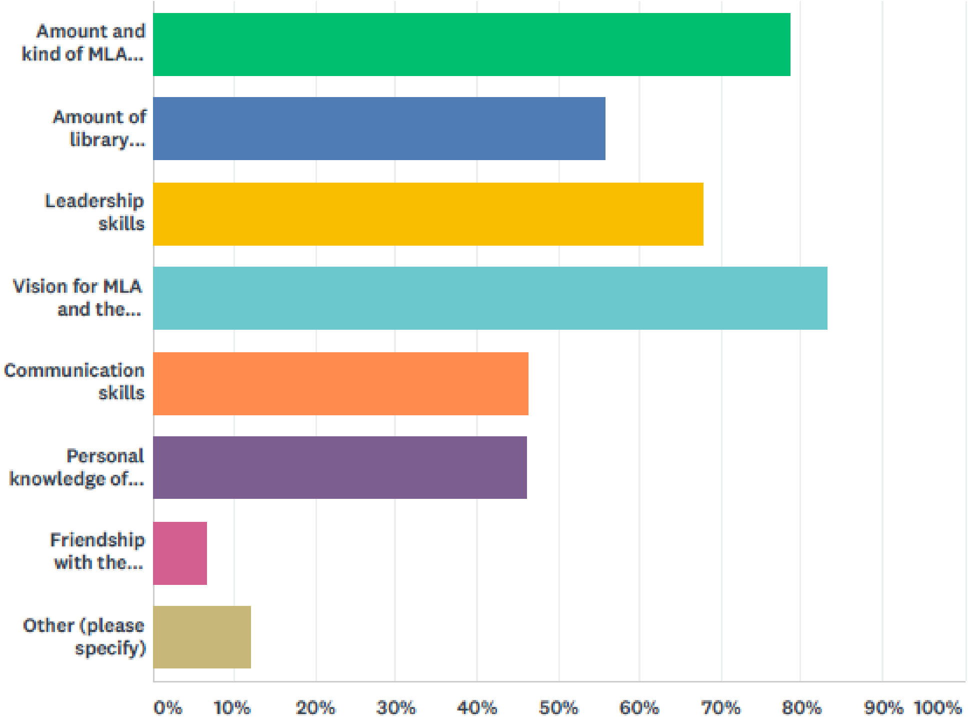

Answer choices	Responses	
Amount and kind of MLA experience (i.e., service on national committees, task forces, etc.; service to MLA sections and chapters; etc.)	78.68%	524
Amount of library experience	55.86%	372
Leadership skills	67.72%	451
Vision for MLA and the profession expressed through candidate's statement, publications, presentations, etc.	83.03%	553
Communication skills	46.25%	308
Personal knowledge of the candidate	45.95%	306
Friendship with the candidate	6.76%	45
Other (please specify)	11.86%	79
Total		666

JMLA Journal of the Medical Library Association

SC
Supplemental content to
J Med Libr Assoc. Jul;108(3):dx.doi.org/10.5195/jmla.2020.480
www.jmla.mlanet.org
© Shedlock, McQuillen 2020

Q13 What qualities do you look for when voting for MLA Board of Directors? Check all that apply.
Answered: 654 Skipped: 22

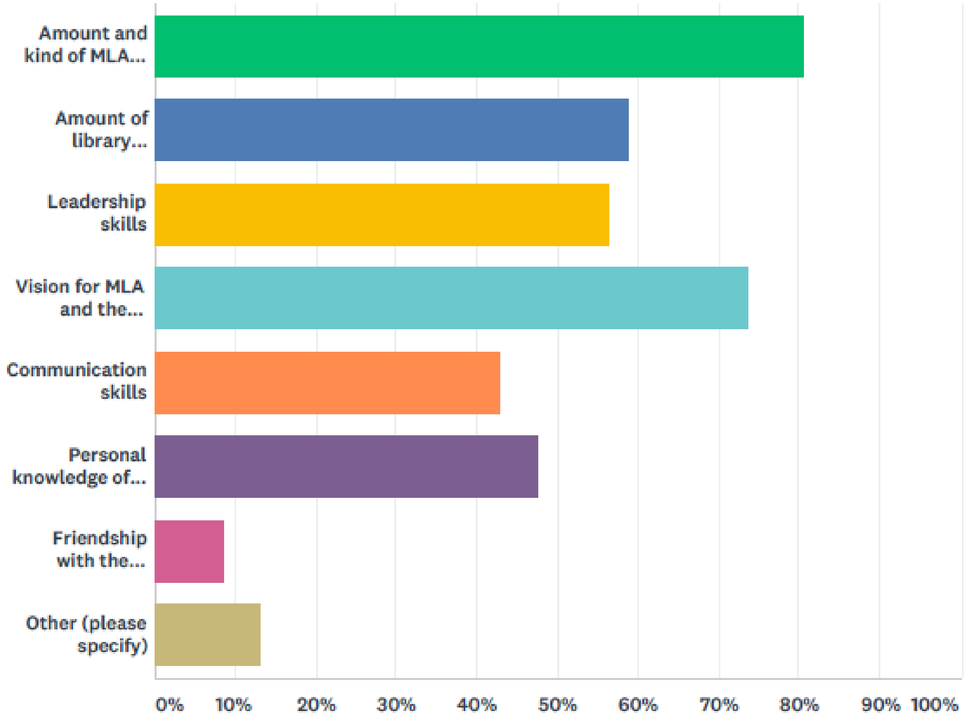

Answer choices	Responses	
Amount and kind of MLA experience (i.e., service on national committees, task forces, etc.; service to MLA sections and chapters; etc.)	80.58%	527
Amount of library experience	58.87%	385
Leadership skills	56.57%	370
Vision for MLA and the profession expressed through candidate's statement, publications, presentations, etc.	73.70%	482
Communication skills	42.81%	280
Personal knowledge of the candidate	47.40%	310
Friendship with the candidate	8.56%	56
Other (please specify)	13.15%	86
Total		654

Q14 What qualities do you look for when voting for MLA Nominating Committee? Check all that apply.
Answered: 643 Skipped: 33

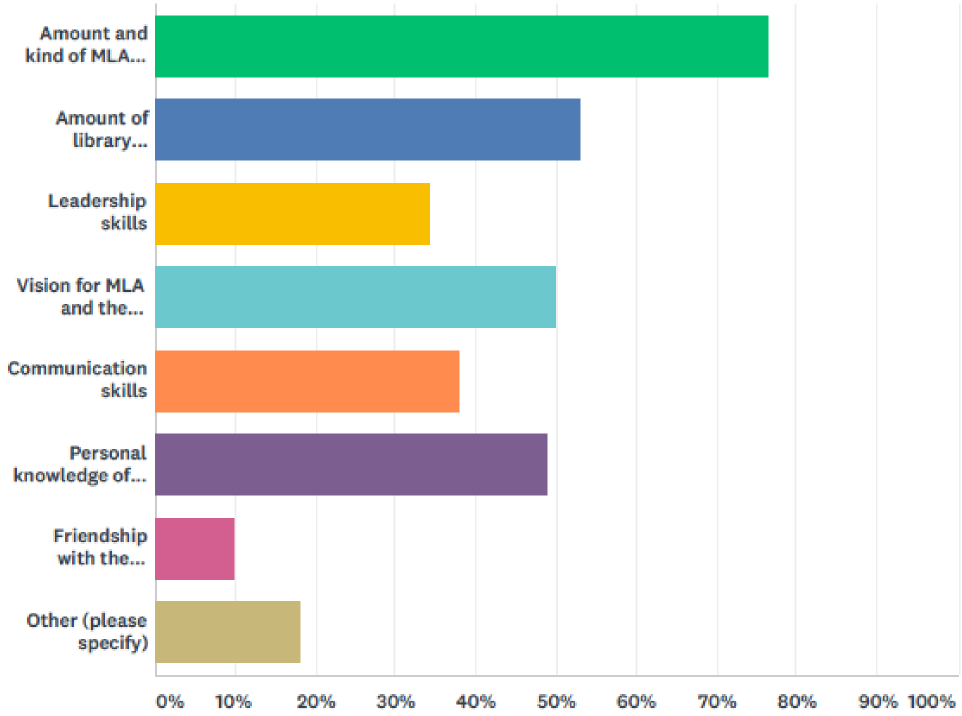

Answer choices	Responses	
Amount and kind of MLA experience (i.e., service on national committees, task forces, etc.; service to MLA sections and chapters; etc.)	76.67%	493
Amount of library experience	53.03%	341
Leadership skills	34.21%	220
Vision for MLA and the profession expressed through candidate's statement, publications, presentations, etc.	49.92%	321
Communication skills	37.95%	244
Personal knowledge of the candidate	48.99%	315
Friendship with the candidate	9.80%	63
Other (please specify)	18.04%	116
Total		643

Journal of the Medical Library Association

Supplemental content to
J Med Libr Assoc. Jul;108(3):dx.doi.org/10.5195/jmla.2020.480
www.jmla.mlanet.org
© Shedlock, McQuillen 2020

Q15 Given all the factors above that are important to you when voting for MLA national office (president, Board of Directors, Nominating Committee) and given that these factors are relatively equal among the candidates, do you consider any of the following characteristics when making a decision for whom to vote?

Answered: 645 Skipped: 31

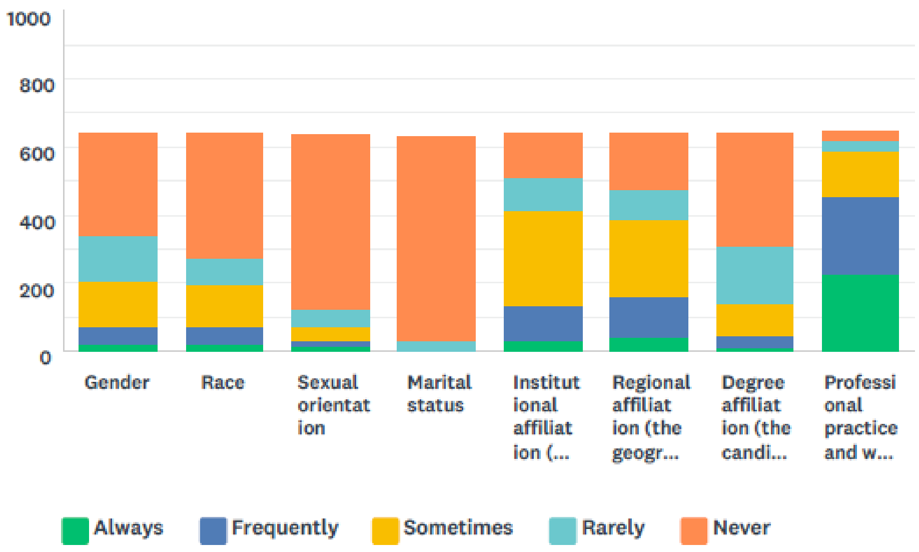

	Always		Frequently		Sometimes		Rarely		Never		Total respondents
	%	n	%	n	%	n	%	n	%	n	
Gender	2.97%	19	7.66%	49	21.72%	139	20.47%	131	47.34%	303	640
Race	3.12%	20	7.79%	50	19.16%	123	12.31%	79	58.26%	374	642
Sexual orientation	2.37%	15	2.37%	15	6.31%	40	8.04%	51	81.07%	514	634
Marital status	–	0	0.16%	1	0.32%	2	4.60%	29	95.09%	600	631

JMLA Journal of the Medical Library Association

SC
Supplemental content to
J Med Libr Assoc. Jul;108(3):dx.doi.org/10.5195/jmla.2020.480
www.jmla.mlanet.org
© Shedlock, McQuillen 2020

	**Always**		**Frequently**		**Sometimes**		**Rarely**		**Never**		**Total respondents**
	**%**	**n**	**%**	**n**	**%**	**n**	**%**	**n**	**%**	**n**	
Institutional affiliation (the candidate's employer)	4.70%	30	16.30%	104	43.42%	277	15.83%	101	20.53%	131	638
Regional affiliation (the geographic area where the candidate works or is from)	6.25%	40	18.44%	118	35.16%	225	14.69%	94	26.56%	170	640
Degree affiliation (the candidate's alma mater)	1.41%	9	5.49%	35	15.07%	96	26.69%	170	52.59%	335	637
Professional practice and work history (the candidate's predominant practice field; i.e., public services, technical services, media services, technology services, administrative services, etc.)	34.57%	223	34.88%	225	20.93%	135	5.12%	33	5.43%	35	645

Supplemental content to
J Med Libr Assoc. Jul;108(3):dx.doi.org/10.5195/jmla.2020.480
www.jmla.mlanet.org
© Shedlock, McQuillen 2020

Q16 Given all the factors above—professional and personal—what is the one or most important deciding factor when casting your vote for MLA president?

Answered: 545 Skipped: 131

Q17 Given all the factors above—professional and personal—what is the one or most important deciding factor when casting your vote for MLA Board of Directors?

Answered: 539 Skipped: 137

Q18 Given all the factors above—professional and personal—what is the one or most important deciding factor when casting your vote for a candidate for Nominating Committee?

Answered: 531 Skipped: 145

Q19 Do you believe MLA should use a single slate for president and Board of Directors (i.e., rely on the elected Nominating Committee to find the right leadership for MLA)?

Answered: 636 Skipped: 40

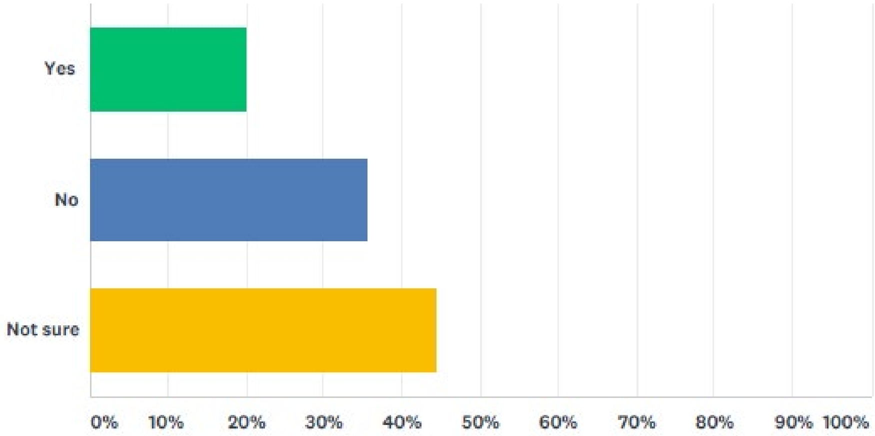

Answer choices	Responses	
Yes	20.13%	128
No	35.53%	226
Not sure	44.34%	282
Total		636

SC
Supplemental content to
J Med Libr Assoc. Jul;108(3):dx.doi.org/10.5195/jmla.2020.480
www.jmla.mlanet.org
© Shedlock, McQuillen 2020

Q20 What is your view of single slates for sections and/or chapters?
Answered: 573 Skipped: 103

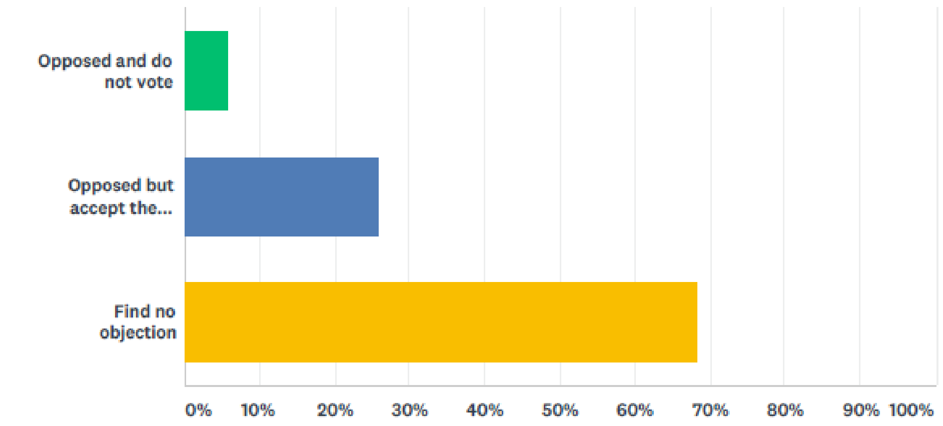

Answer choices	Responses	
Opposed and do not vote	5.93%	34
Opposed but accept the practice	25.83%	148
Find no objection	68.24%	391
Total		573

Q21 How important is it to vote every year for MLA leadership positions (president, Board of Directors, Nominating Committee)?
Answered: 626 Skipped: 50

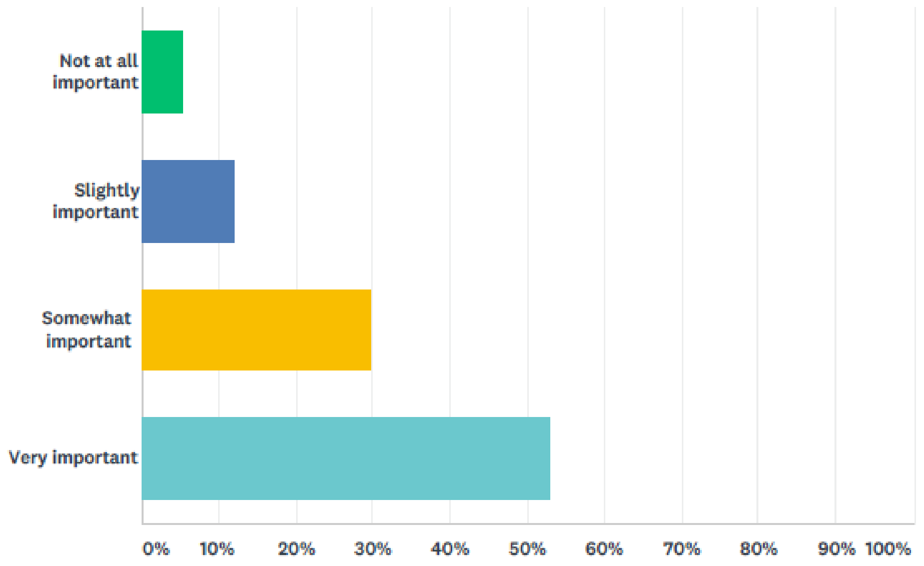

JMLA Journal of the Medical Library Association

SC
Supplemental content to
J Med Libr Assoc. Jul;108(3):dx.doi.org/10.5195/jmla.2020.480
www.jmla.mlanet.org
© Shedlock, McQuillen 2020

Answer choices	Responses	
Not at all important	5.43%	34
Slightly important	11.98%	75
Somewhat important	29.71%	186
Very important	52.88%	331
Total		626

Q22 Mark the statement most true to your experience:
Answered: 626 Skipped: 50

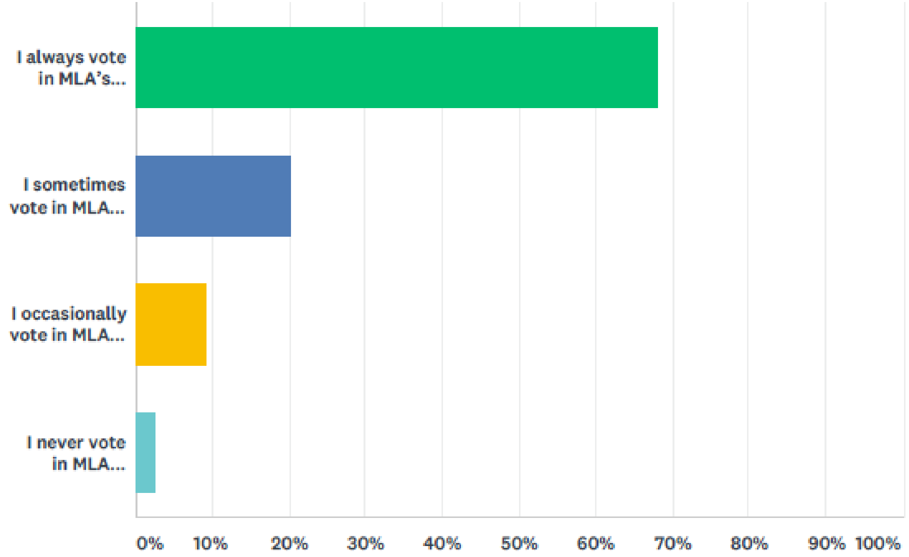

Answer choices	Responses	
I always vote in MLA's national election.	67.89%	425
I sometimes vote in MLA elections (3 or more times out of every 5 elections).	20.29%	127
I occasionally vote in MLA elections (2 or fewer times out of 5 elections).	9.27%	58
I never vote in MLA elections.	2.56%	16
Total		626

Q23 If you haven't voted in most MLA national elections, what would encourage you to vote in an MLA national election?
Answered: 129 Skipped: 547

JMLA Journal of the Medical Library Association

SC
Supplemental content to
J Med Libr Assoc. Jul;108(3):dx.doi.org/10.5195/jmla.2020.480
www.jmla.mlanet.org
© Shedlock, McQuillen 2020

Q24 Do you consider voting in MLA elections a membership benefit?
Answered: 622 Skipped: 54

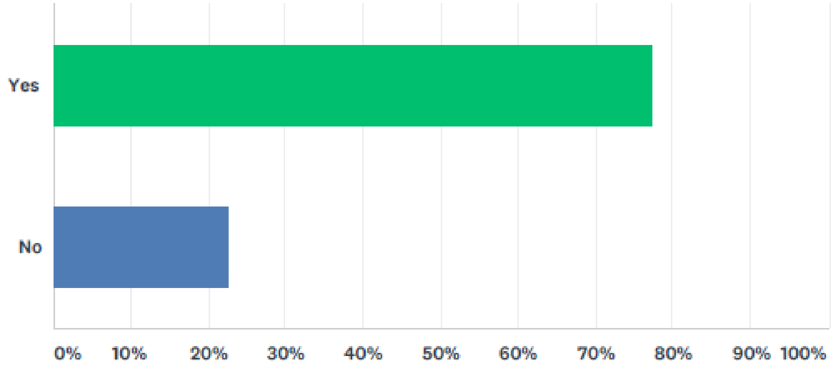

Answer choices	Responses	
Yes	77.49%	482
No	22.51%	140
Total		622

Q25 Do you consider voting in MLA elections a membership responsibility?
Answered: 623 Skipped: 53

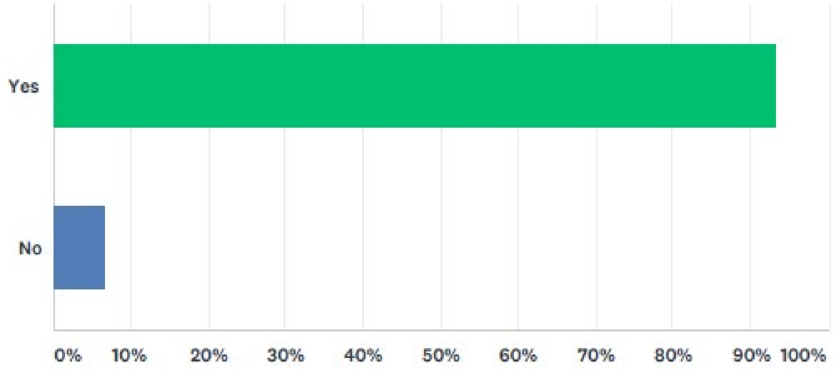

Answer choices	Responses	
Yes	93.26%	581
No	6.74%	42
Total		623

Supplemental content to
J Med Libr Assoc. Jul;108(3):dx.doi.org/10.5195/jmla.2020.480
www.jmla.mlanet.org
© Shedlock, McQuillen 2020

Q26 If you have both individual/personal and institutional memberships, do you ever split your vote among candidates (i.e., use personal membership to vote for one candidate and institutional membership to vote for another candidate)?

Answered: 612 Skipped: 64

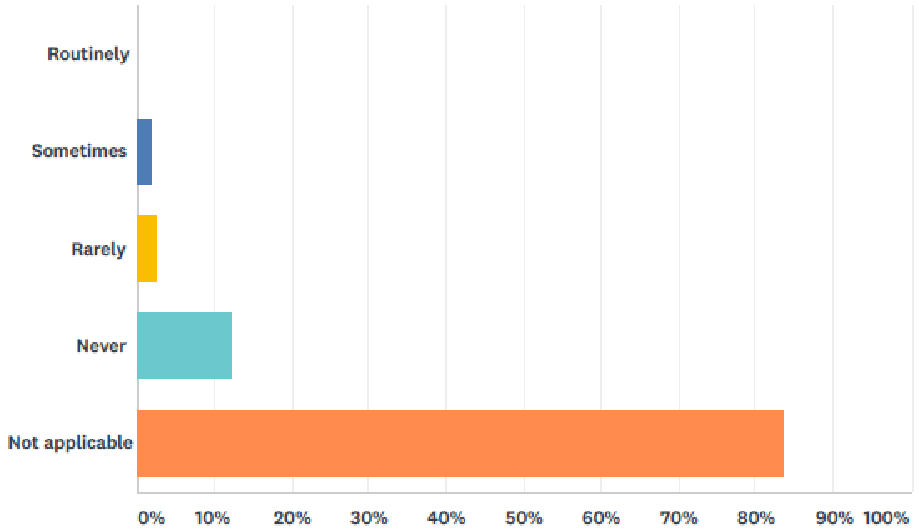

Answer choices	Responses	
Routinely	–	0
Sometimes	1.80%	11
Rarely	2.45%	15
Never	12.09%	74
Not applicable	83.66%	512
Total		612

Q27 Have you ever nominated a person for MLA office?

Answered: 623 Skipped: 53

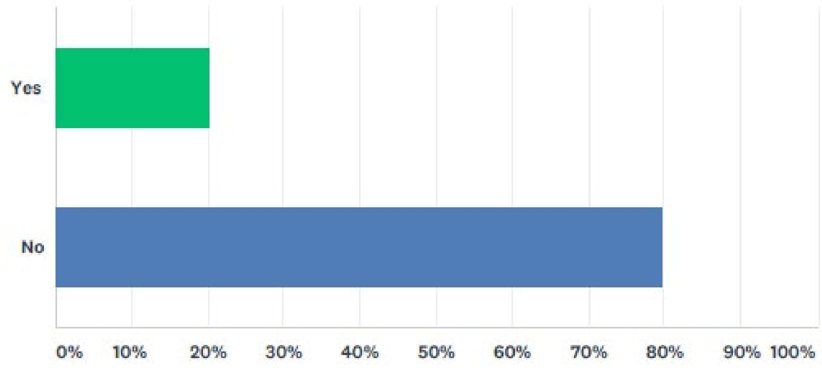

SC
Supplemental content to
J Med Libr Assoc. Jul;108(3):dx.doi.org/10.5195/jmla.2020.480
www.jmla.mlanet.org
© Shedlock, McQuillen 2020

Answer choices	Responses	
Yes	20.22%	126
No	79.78%	497
Total		623

Q28 If Yes was marked in Q 27, was the person selected as a candidate?
Answered: 134 Skipped: 542

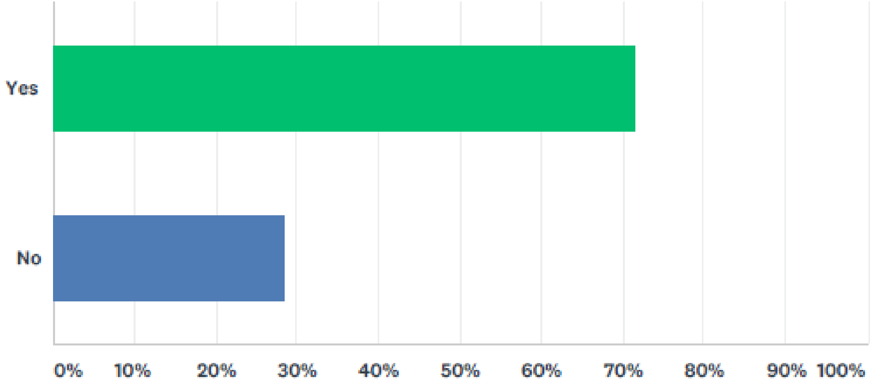

Answer choices	Responses	
Yes	71.64%	96
No	28.36%	38
Total		134

Q29 Have you ever nominated yourself for MLA office?
Answered: 611 Skipped: 65

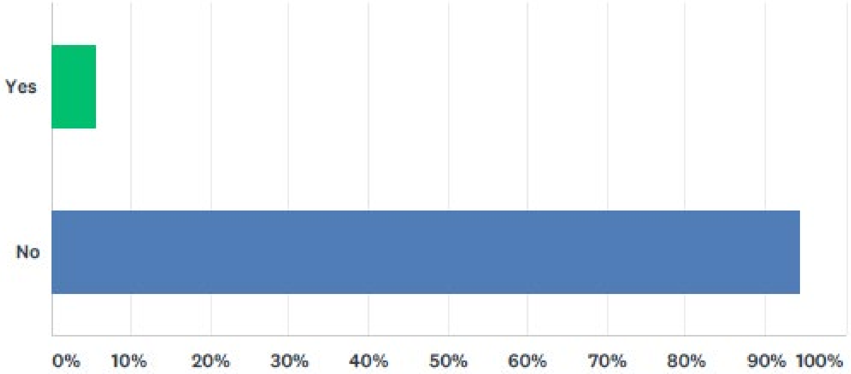

Supplemental content to
J Med Libr Assoc. Jul;108(3):dx.doi.org/10.5195/jmla.2020.480
www.jmla.mlanet.org
© Shedlock, McQuillen 2020

Answer choices	Responses	
Yes	5.56%	34
No	94.44%	577
Total		611

Q30 If Yes was selected in Q 29, were you selected as a candidate?

Answered: 54 Skipped: 622

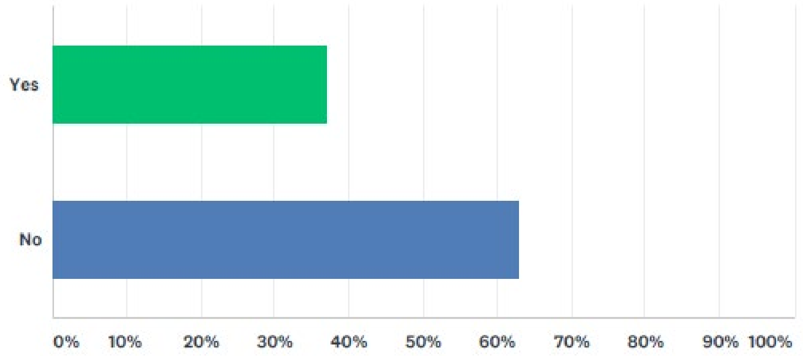

Answer choices	Responses	
Yes	37.04%	20
No	62.96%	34
Total		54

Q31 Have you ever "campaigned" on behalf of an MLA nominee? Campaigning is defined here as conversing with MLA members inside your library, in your geographic region or chapter, in a section, among friends in MLA, etc., for the purpose of persuading a vote on behalf of or against an MLA nominee. Conversing may be in person, by phone, via email, or via other social media.

Answered: 620 Skipped: 56

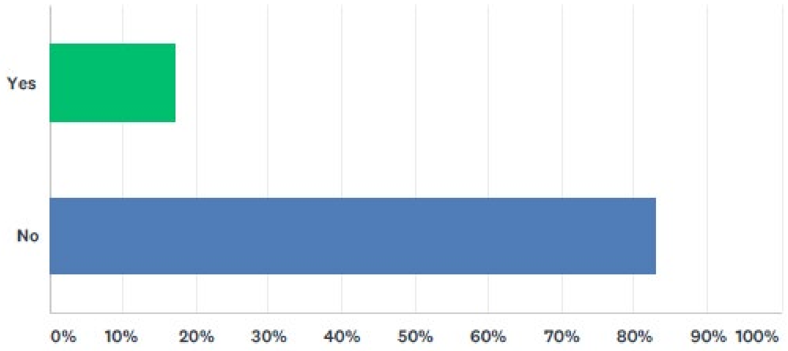

Supplemental content to
J Med Libr Assoc. Jul;108(3):dx.doi.org/10.5195/jmla.2020.480
www.jmla.mlanet.org
© Shedlock, McQuillen 2020

Answer choices	Responses	
Yes	17.10%	106
No	82.90%	514
Total		620

Q32 Traditionally, MLA has not engaged in campaign activities for national office. Do you agree with this tradition?

Answered: 622 Skipped: 54

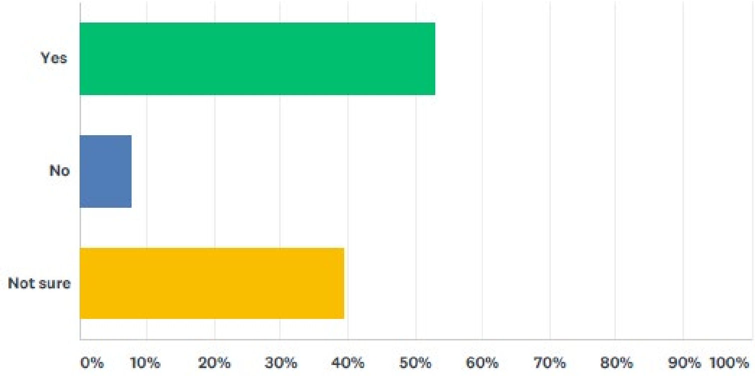

Answer choices	Responses	
Yes	52.89%	329
No	7.72%	48
Not sure	39.39%	245
Total		622

Q33 Do you think campaigning would have a positive or negative impact on MLA elections?

Answered: 620 Skipped: 56

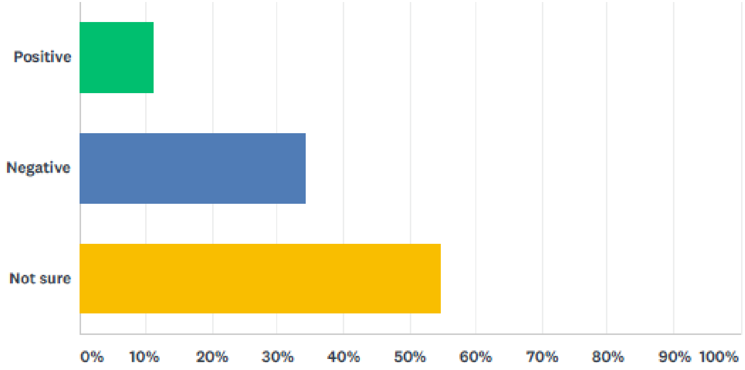

JMLA Journal of the Medical Library Association

SC
Supplemental content to
J Med Libr Assoc. Jul;108(3):dx.doi.org/10.5195/jmla.2020.480
www.jmla.mlanet.org
© Shedlock, McQuillen 2020

Answer choices	Responses	
Positive	11.13%	69
Negative	34.35%	213
Not sure	54.52%	338
Total		620

Q34 Do you think the MLA Board should define controls for campaigning in MLA national elections?
Answered: 608 Skipped: 68

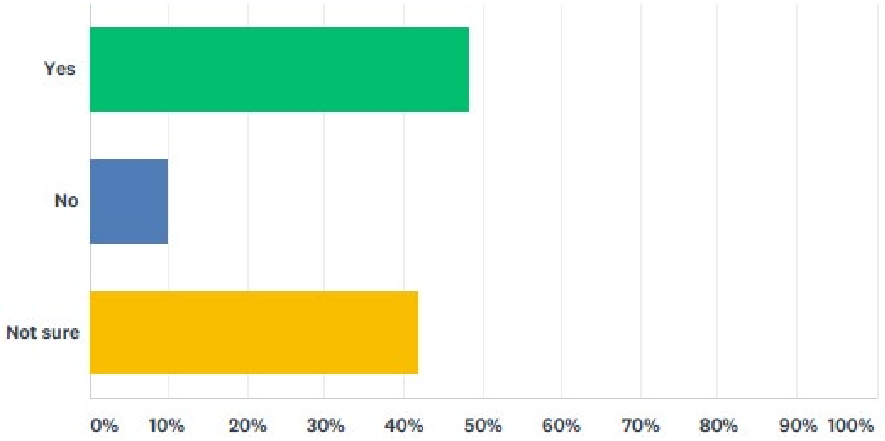

Answer choices	Responses	
Yes	48.36%	294
No	9.87%	60
Not sure	41.78%	254
Total		608

Supplemental content to
J Med Libr Assoc. Jul;108(3):dx.doi.org/10.5195/jmla.2020.480
www.jmla.mlanet.org
© Shedlock, McQuillen 2020

Q35 How useful are candidate's statements in your decision-making process?

Answered: 616 Skipped: 60

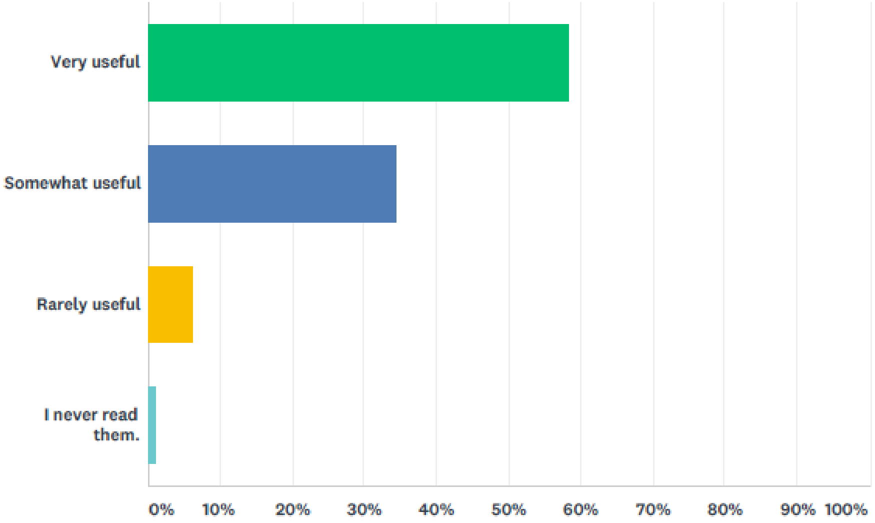

Answer choices	Responses	
Very useful	58.28%	359
Somewhat useful	34.42%	212
Rarely useful	6.33%	39
I never read them	0.97%	6
Total		616

Q36 How useful is MLA's Nominating Committee process (i.e., electing a Nominating Committee to create a slate of candidates for president and Board of Directors and asking the candidates questions about their views for the office they agree to seek)?

Answered: 604 Skipped: 72

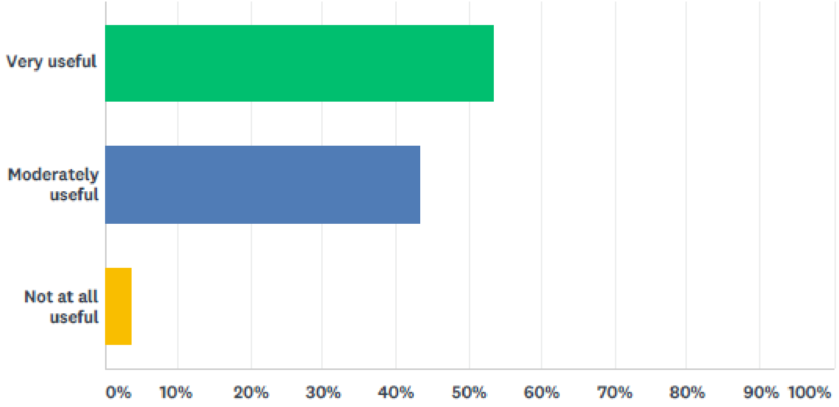

JMLA Journal of the Medical Library Association

SC
Supplemental content to
J Med Libr Assoc. Jul;108(3):dx.doi.org/10.5195/jmla.2020.480
www.jmla.mlanet.org
© Shedlock, McQuillen 2020

Answer choices	Responses	
Very useful	53.31%	322
Moderately useful	43.21%	261
Not at all useful	3.48%	21
Total		604

Q37 Have you considered nominating an additional candidate to the Nominating Committee's slate (i.e., using the nomination by petition process)?

Answered: 615 Skipped: 61

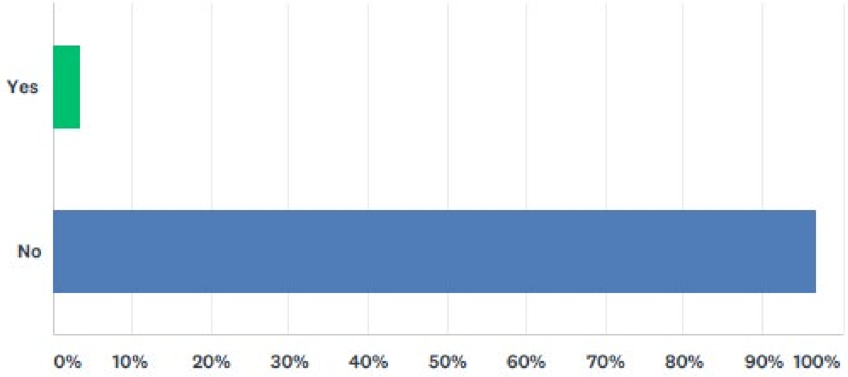

Answer choices	Responses	
Yes	3.25%	20
No	96.75%	595
Total		615

Q38 Would you consider using the nomination by petition process in the future?

Answered: 585 Skipped: 91

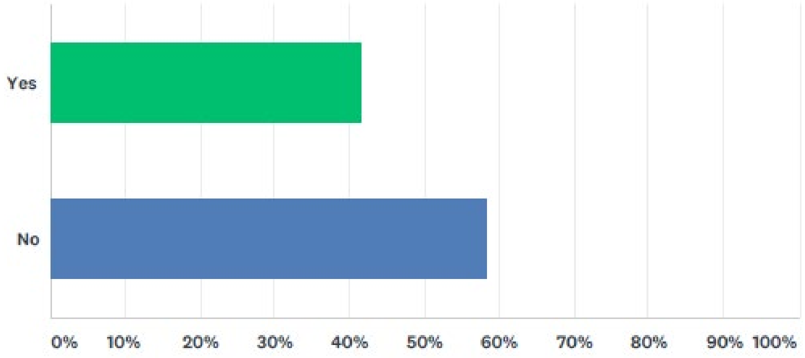

Supplemental content to
J Med Libr Assoc. Jul;108(3):dx.doi.org/10.5195/jmla.2020.480
www.jmla.mlanet.org
© Shedlock, McQuillen 2020

Answer choices	Responses	
Yes	41.54%	243
No	58.46%	342
Total		585

Q39 In your opinion what factor(s) gives an MLA candidate an advantage over another candidate?
Answered: 482 Skipped: 194

Q40 How often do you attend MLA annual meetings?
Answered: 617 Skipped: 59

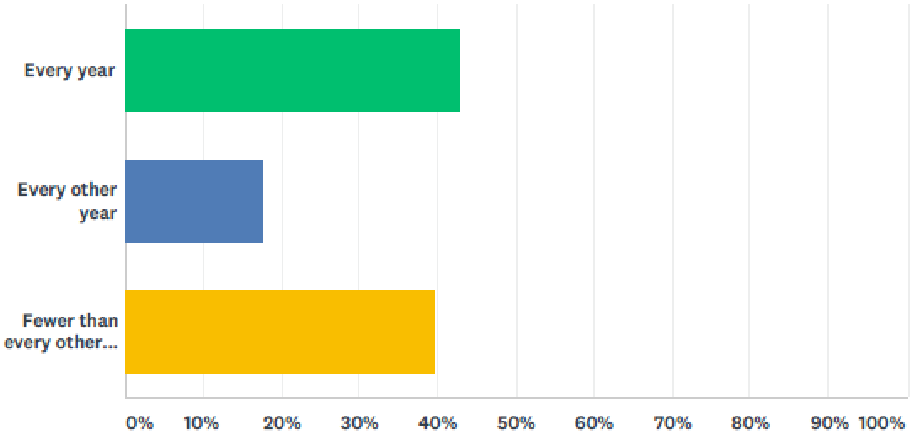

Answer choices	Responses	
Every year	42.95%	265
Every other year	17.50%	108
Fewer than every other year	39.55%	244
Total		617

J Med Libr Assoc. Jul;108(3):dx.doi.org/10.5195/jmla.2020.480
www.jmla.mlanet.org
© Shedlock, McQuillen 2020

Q41 How often do you attend chapter meetings?
Answered: 607 Skipped: 69

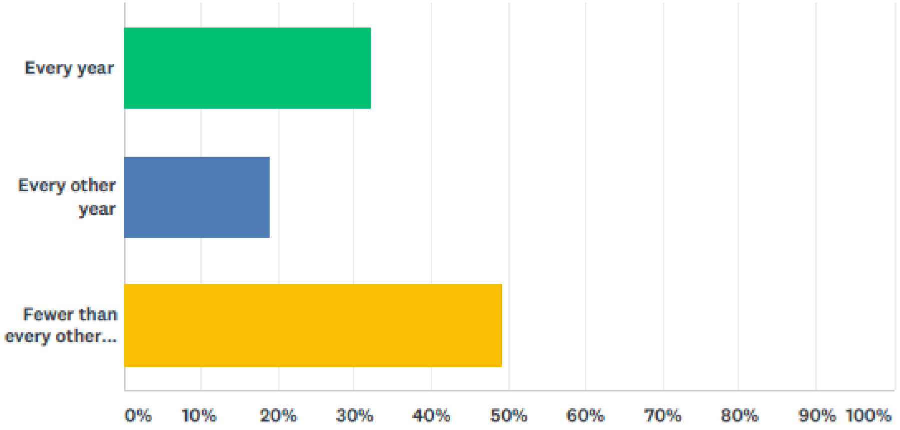

Answer choices	Responses	
Every year	31.96%	194
Every other year	18.78%	114
Fewer than every other year	49.26%	299
Total		607

Q42 Are you currently on or have you ever served on a national committee, editorial board, ad hoc group, task force, jury, or other national body, or written for a national publication (*JMLA/BMLA, MLA News*)?
Answered: 617 Skipped: 59

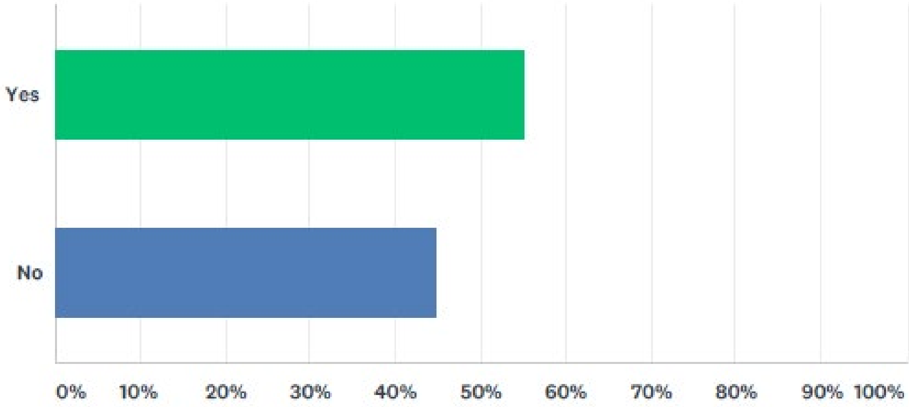

JMLA Journal of the Medical Library Association

SC
Supplemental content to
J Med Libr Assoc. Jul;108(3):dx.doi.org/10.5195/jmla.2020.480
www.jmla.mlanet.org
© Shedlock, McQuillen 2020

Answer choices	Responses	
Yes	55.27%	341
No	44.73%	276
Total		617

Q43 Are you currently on or have you ever served your MLA chapter via holding an elected office; serving on a chapter committee; having been appointed to a committee, task force, group, or other body; or having written for a chapter publication, etc.?
Answered: 610 Skipped: 66

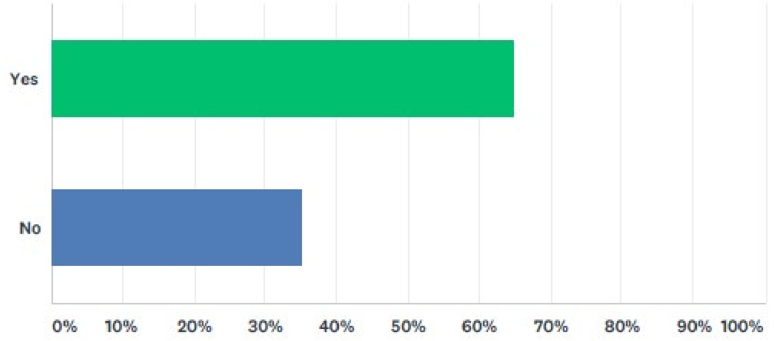

Answer choices	Responses	
Yes	64.92%	396
No	35.08%	214
Total		610

Q44 Are you currently on or have you ever served an MLA section via holding an elected office; serving on a section committee; having been appointed to a committee, task force, group, or other body; or having written for a section publication; etc.?
Answered: 611 Skipped: 65

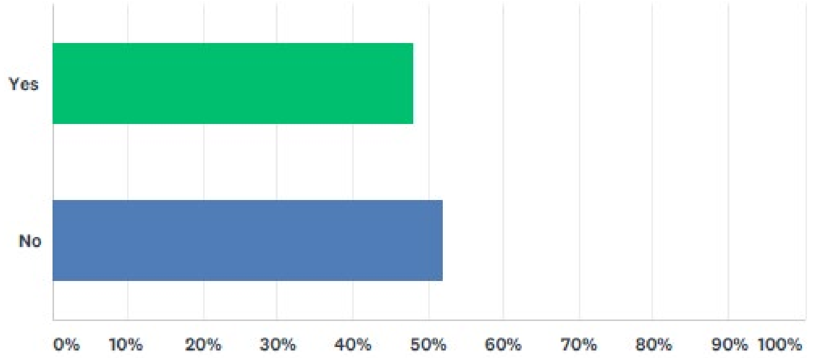

JMLA Journal of the Medical Library Association

Supplemental content to
J Med Libr Assoc. Jul;108(3):dx.doi.org/10.5195/jmla.2020.480
www.jmla.mlanet.org
© Shedlock, McQuillen 2020

Answer choices	Responses	
Yes	48.12%	294
No	51.88%	317
Total		611

Q45 Please offer any other ways you feel connected to MLA, its chapters, and its sections.
Answered: 199 Skipped: 477

Q46 Are there other issues related to the MLA voting process that should be considered?
Answered: 96 Skipped: 580
