# Supplementary material for: The Medical Library Association (MLA) voter: a survey of attitudes, perceptions, and voting practices in MLA national elections
Source: J Med Libr Assoc. 2020 Jul 1;108(3):452–62. doi: 10.5195/jmla.2020.480 (PMC7441894; doi:10.5195/jmla.2020.480)
Supplement: Supplementary file 4 — Appendix D: Respondents' geographic location by Medical Library Association chapter [file jmla-108-3-452-s04.pdf]

JMLA Journal of the Medical Library Association

Supplemental content to
J Med Libr Assoc. Jul;108(3):dx.doi.org/10.5195/jmla.2020.480
www.jmla.mlanet.org
© Shedlock, McQuillen 2020

The Medical Library Association (MLA) voter: a survey of attitudes, perceptions, and voting practices in MLA national elections

James Shedlock, AMLS, AHIP, FMLA; Elizabeth Perkin McQuillen, PhD

APPENDIX D

Respondents' geographic location by Medical Library Association chapter

MLA chapter	Chapter membership 2017	Number of respondents	Respondents as a percentage of chapter membership	Percent of survey respondents
Midwest Chapter	278	128	46.04%	19.19%
Mid-Atlantic Chapter (MAC)	231	96	41.55%	14.39%
South Central Chapter (SCC)	271	80	29.52%	11.99%
Southern Chapter	313	66	21.08%	9.89%
Midcontinental Chapter (MCMLA)	103	55	53.34%	8.25%
Medical Library Group of Southern California Arizona (MLGSCA)	NA	54	NA	8.10%
North Atlantic Health Sciences Libraries (NAHSL)	143	40	27.97%	6.0%
New York-New Jersey Chapter (NY-NJ)	176	39	22.16%	5.85%
Pacific Northwest Chapter of MLA	64	24	37.50%	3.60%
Northern California and Nevada Medical Library Group (NCNMLG)	NA	24	NA	3.60%
Upstate New York and Ontario Chapter (UNYOC)	90	22	24.44%	3.30%

JMLA Journal of the Medical Library Association

Supplemental content to
J Med Libr Assoc. Jul;108(3):dx.doi.org/10.5195/jmla.2020.480
www.jmla.mlanet.org
© Shedlock, McQuillen 2020

MLA chapter	Chapter membership 2017	Number of respondents	Respondents as a percentage of chapter membership	Percent of survey respondents
Philadelphia Regional Chapter	67	20	29.85%	3.0%
Canada	NA	18	NA	2.70%
Hawaii-Pacific Chapter	21	1	0.05%	0.15%
Other country	NA	4	NA	0.60%
